# Supplementary material for: The LINC01612-DVL2-WNT axis promotes human endoderm differentiation
Source: Stem Cell Reports. 2025 Oct 23;20(11):102682. doi: 10.1016/j.stemcr.2025.102682 (PMC12790727; doi:10.1016/j.stemcr.2025.102682)
Supplement: Document S1. Figures S1–S6, Tables S1 and S5, and supplemental methods [file mmc1.pdf]

Stem Cell Reports, Volume *20*

Supplemental Information

The *LINC01612*-DVL2-WNT axis promotes human endoderm differentiation

Mao Li, Pei Lu, Jie Yang, Chenchao Yan, Yikang Yang, and Wei Jiang

Supplemental Information

Supplementary figures and legends

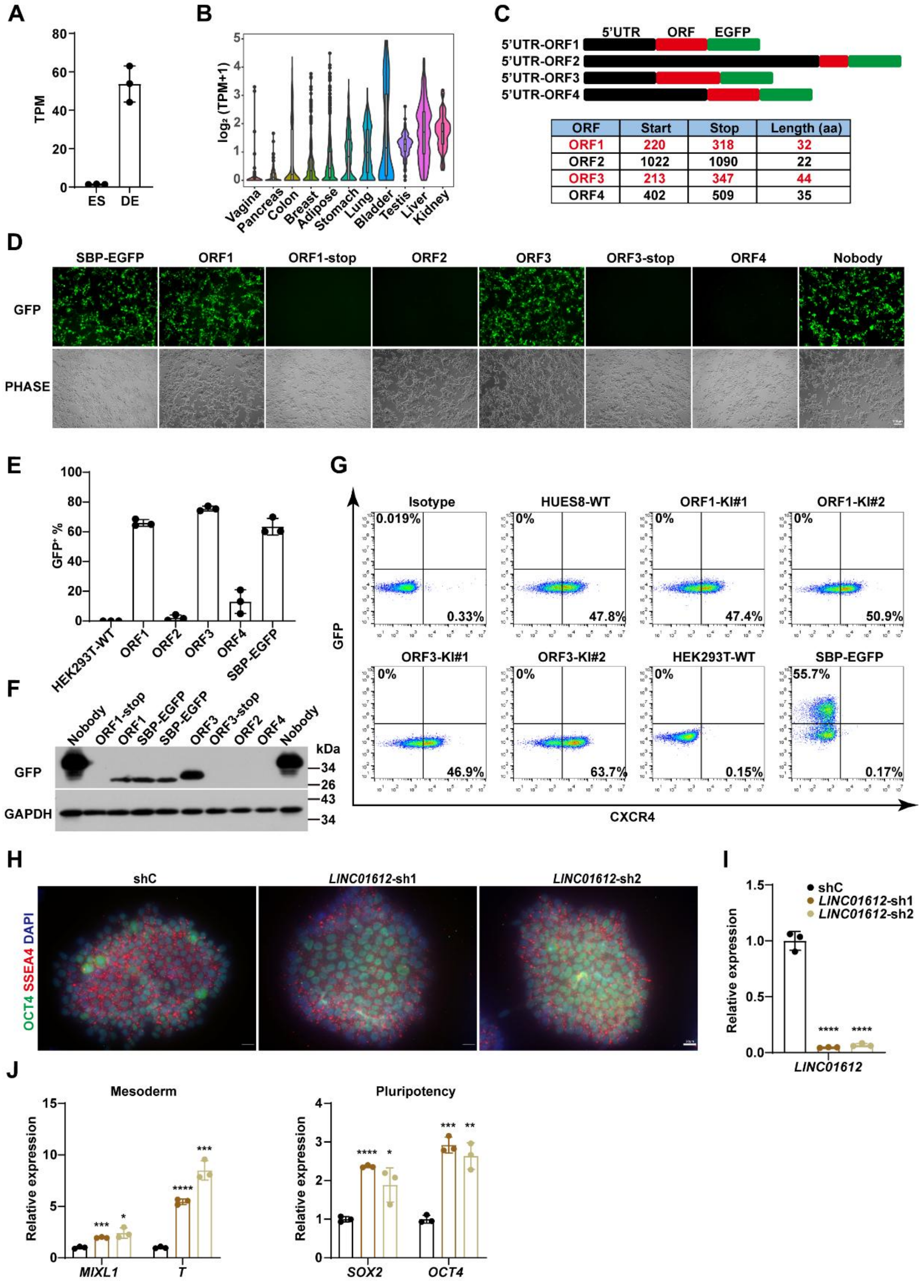

Figure S1. ***LINC01612*** **lacks protein-coding ability, related to Fig 1.**

(A) The TPM value of *LINC01612* in ESC and DE cells (date from GSE162649).

(B) The expression of *LINC01612* in 30 human tissues from GTEx database. The top eleven tissues with high expression were shown. *LINC01612* is merely expressed in the other 29 tissues (Blood, Brain,

Prostate, etc.).

(C) Predicted ORFs of *LINC01612* and diagram of the ORFs constructs with C-terminal EGFP-tag.

(D-E) Expression of ORFs-EGFP of *LINC01612* in HEK293T cells, detected by immunofluorescent staining (D) and flow cytometric analysis (E) (n = 3 independent experiments). SBP-EGFP was used as an empty vector for GFP, and Nobody was used as positive micropeptide control. Scale bar, 100 μm.

(F) Western blot analysis showing the GFP protein levels of ORFs-EGFP in HEK293T cells.

(G) Expression of GFP and CXCR4 in ORF1/3 knockin HUES8-DE cells, detected by flow cytometric analysis. SBP-EGFP transfected HEK293T cells was used as a control.

(H) Immunofluorescent staining of pluripotency markers (OCT4 and SSEA4) in *LINC01612*-KD and control ESCs. Scale bar, 20 μm.

(I) Knockdown efficiencies of *LINC01612*-shRNAs in DE cells, examined by RT-qPCR (n = 3 independent experiments).

(J) The RNA levels of marker genes, including mesoderm genes (*MIXL1* and *T*) and pluripotency genes (*SOX2* and *OCT4*), in *LINC01612*-KD and control DE cells (n = 3 independent experiments).

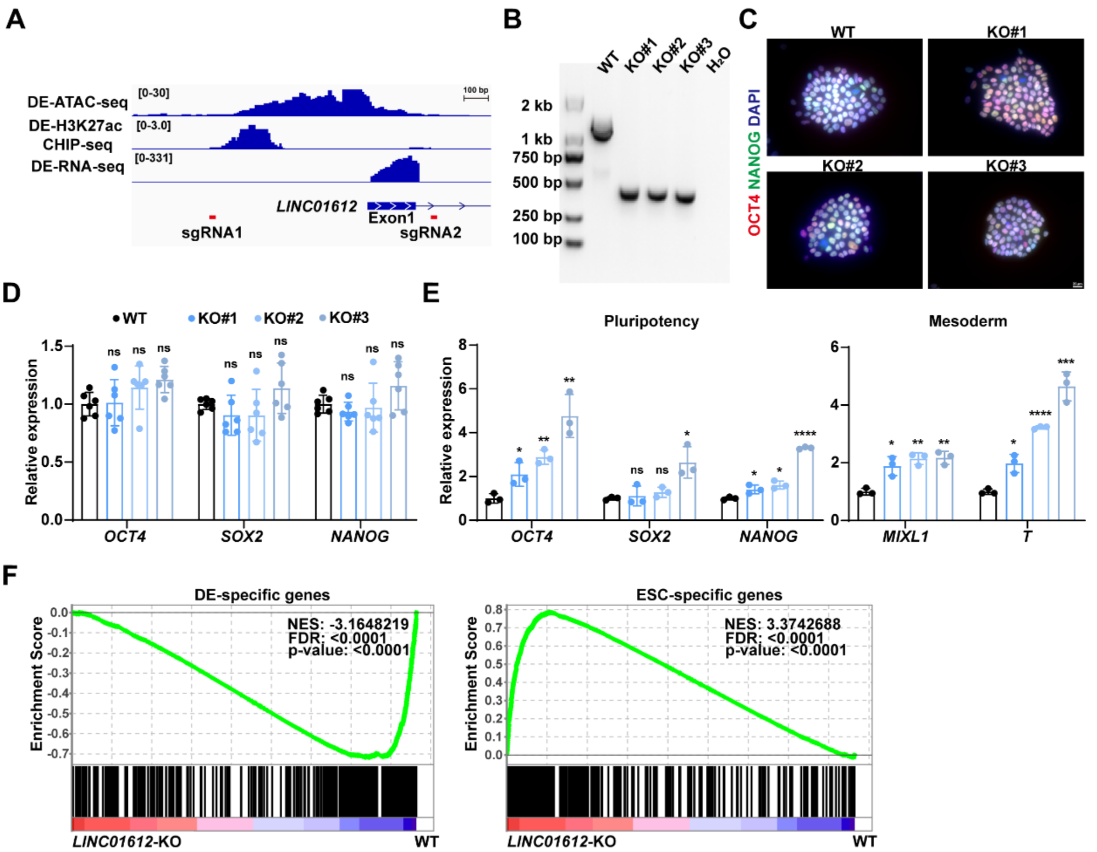

Figure S2. *LINC01612* is not essential for ESC maintenance but required for endoderm differentiation, related to Fig 2.

(A) Bedgraph for ATAC-seq, H3K27ac CHIP-seq and RNA-seq data in DE cells at *LINC01612* promoter loci.

(B) Genomic PCR showing the *LINC01612* deletion in *LINC01612*-KO ESCs.

(C) Immunofluorescent staining of pluripotency markers (OCT4 and NANOG) in wildtype and *LINC01612*- KO ESCs. Scale bar, 20 µm.

(D) The RNA levels of pluripotency genes (*OCT4*, *SOX2* and *NANOG*) in wildtype and *LINC01612*-KO ESCs (n = 6 independent experiments).

(E) The RNA levels of pluripotency genes (*OCT4*, *SOX2* and *NANOG*) and mesoderm genes (*MIXL1* and *T*) in wildtype and *LINC01612*-KO DE cells (n = 3 independent experiments).

(F) GSEA profile of DE-specific genes and ESC-specific genes in wildtype and *LINC01612*-KO DE cells.

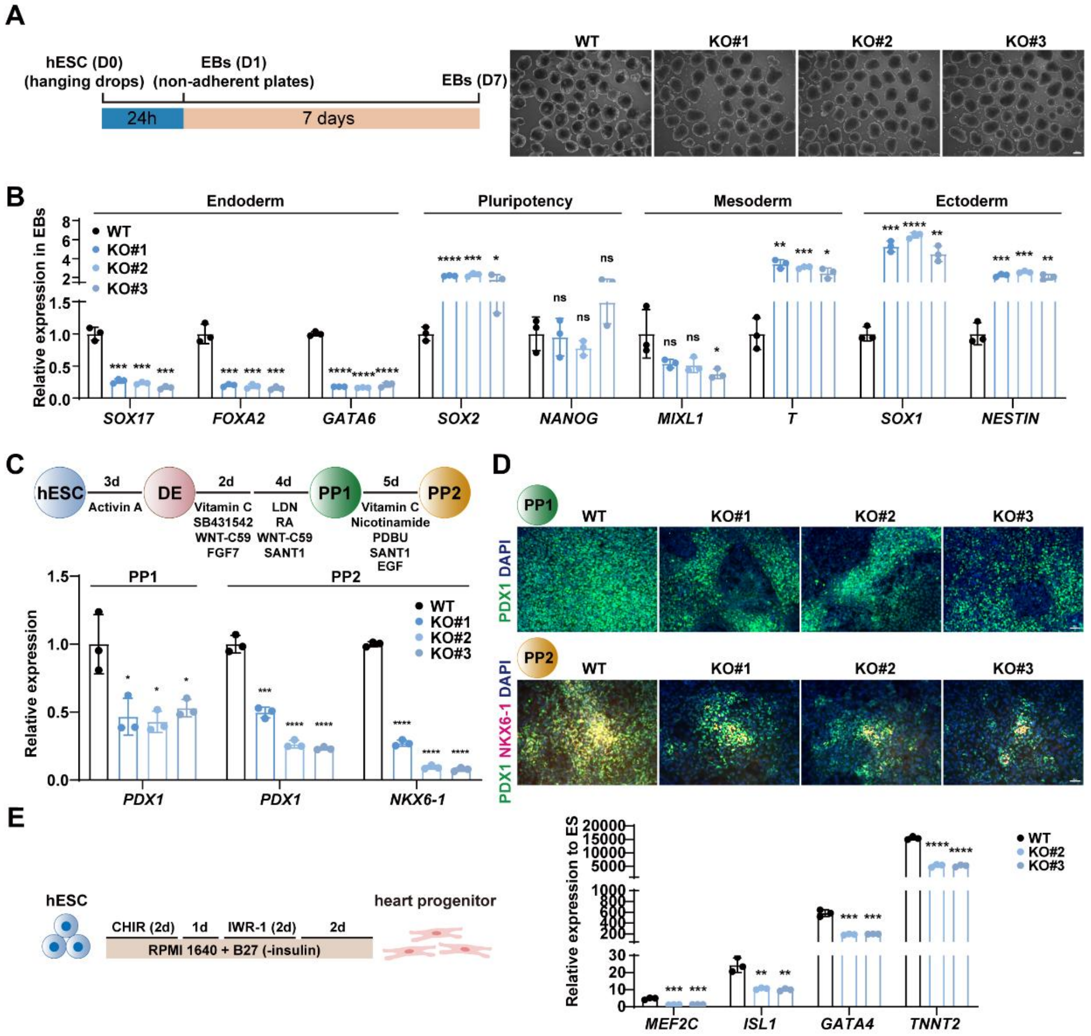

Figure S3. ***LINC01612*** **is required for mesendodermal lineage differentiation, related to Fig 2.**

(A) Left panel: schematic representation of EB differentiation of human ESCs over 7 days. Right panel: bright-field images of WT and *LINC01612*-KO EBs on day 7 in suspension culture, Scale bars, 200 µm.

(B) RT-qPCR analysis of pluripotency markers and lineage-specific markers (endoderm, mesoderm and ectoderm) in wildtype and *LINC01612*-KO EBs on day 7 (n = 3 independent experiments).

(C) Top panel: schematic representation of pancreatic differentiation of human ESCs. Bottom panel: the RNA levels of pancreatic progenitor markers in wildtype and *LINC01612*-KO cells at PP1 and PP2 (n = 3 independent experiments).

(D) Immunofluorescent staining of pancreatic progenitor markers in wildtype and *LINC01612*-KO cells at PP1 and PP2. Scale bars, 50 µm.

(E) Left panel: schematic representation of heart progenitor differentiation of human ESCs. Right panel: the RNA levels of heart progenitor markers in wildtype and *LINC01612*-KO cells (n = 3 independent experiments).

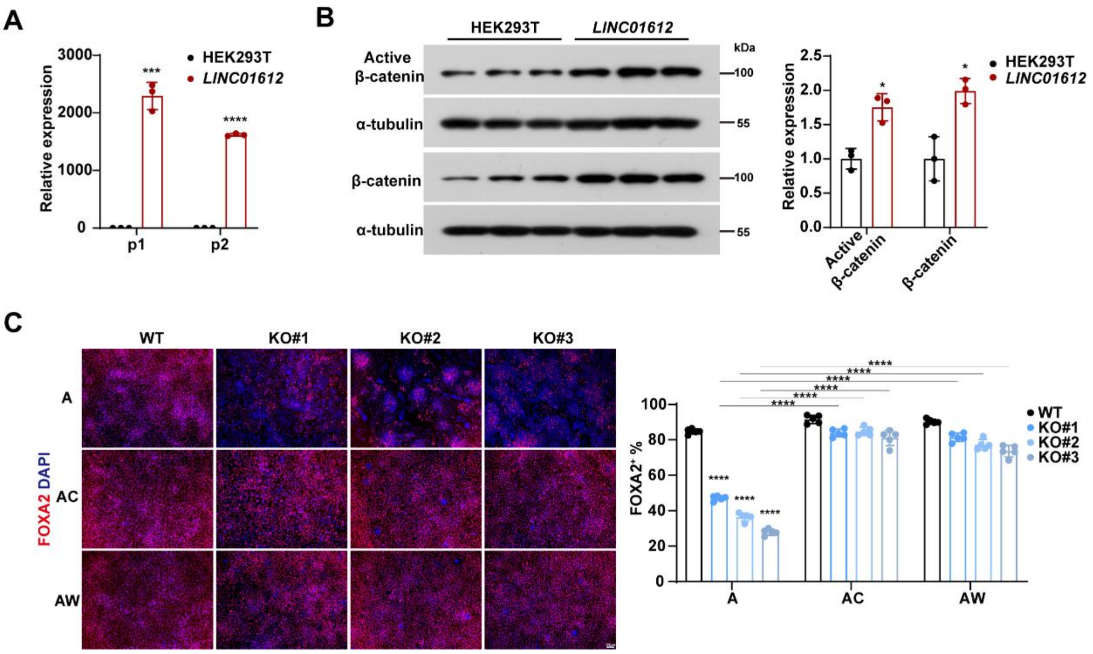

Figure S4. ***LINC01612*** **enhances WNT activity, related to Fig 3.**

(A) Efficient overexpression of *LINC01612* in HEK293T cells (n = 3 independent experiments), p1: primer 1; p2: primer 2.

(B) The levels of active β-catenin and total β-catenin in wildtype and *LINC01612*-overexpressing HEK293T cells. Quantitative results were shown on the right (n = 3 independent experiments).

(C) Immunofluorescent staining detection of FOXA2 in wildtype and *LINC01612*-KO DE cells treated with WNT activators during DE differentiation. Quantitative results were shown on the right (n = 6 images). Scale bar, 100 μm.

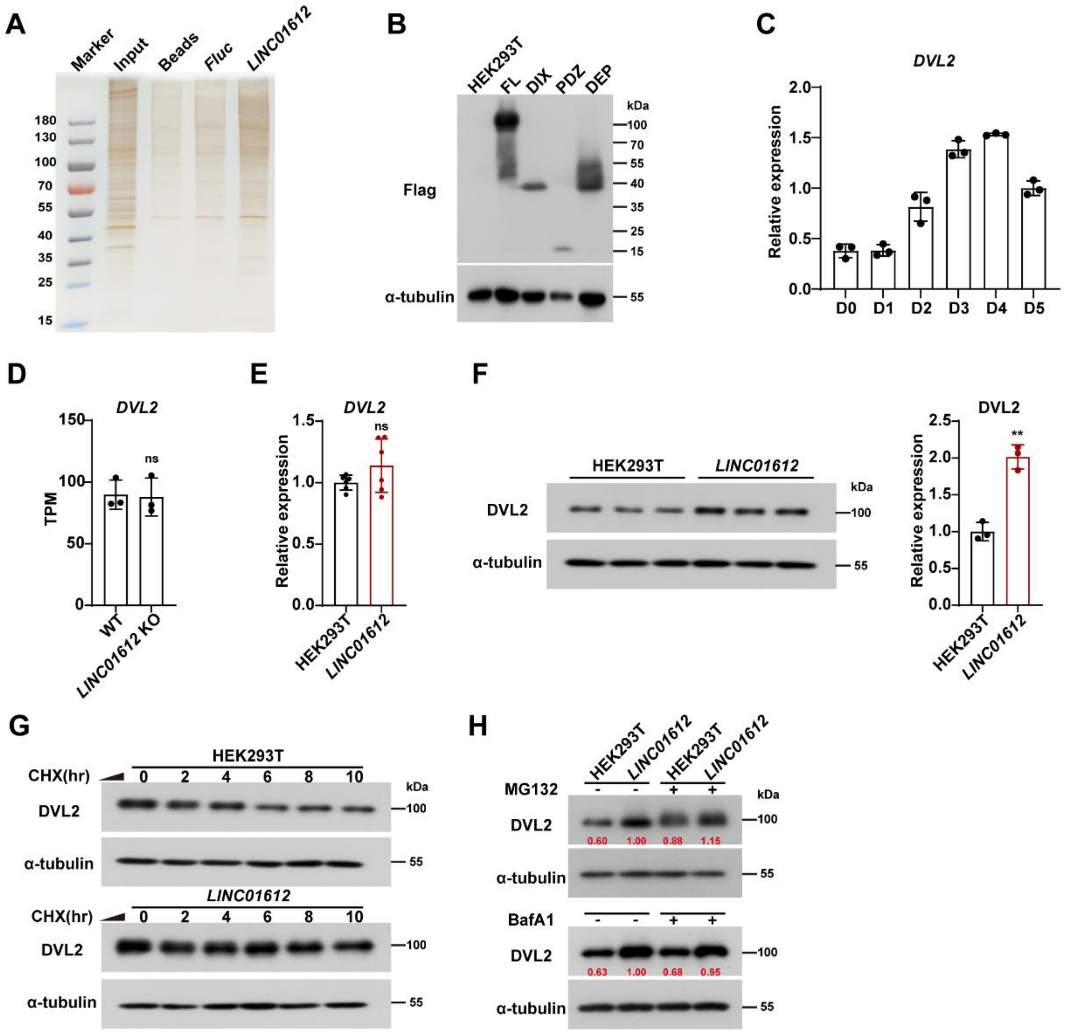

Figure S5. ***LINC01612*** **physically interacts with DVL2 protein and affects its protein stability, related to Fig 4.**

(A) The silver staining results of RNA pulldown in DE cells. Beads and *Fluc* (*Luciferase)* were used as negative controls.

(B) Western blot analysis showing the expression of Flag-tagged full-length DVL2 and its truncations expressed in HEK293T cells.

(C) Time course expression of *DVL2* during human endoderm differentiation detected by RT-qPCR (n = 3 independent experiments).

(D) The TPM value of *DVL2* in wildtype and *LINC01612*-KO DE cells (n = 3 independent experiments).

(E) The RNA level of *DVL2* in wildtype and *LINC01612*-overexpressing HEK293T cells (n = 6 independent experiments).

(F) The protein level of DVL2 in wildtype and *LINC01612*-overexpressing HEK293T cells. Quantitative results were shown on the right (n = 3 independent experiments).

(G) The endogenous protein level of DVL2 in wildtype and *LINC01612*-overexpressing HEK293T cells treated with CHX.

(H) The endogenous protein level of DVL2 in wildtype and *LINC01612*-overexpressing HEK293T cells treated with MG132 or BafA1.

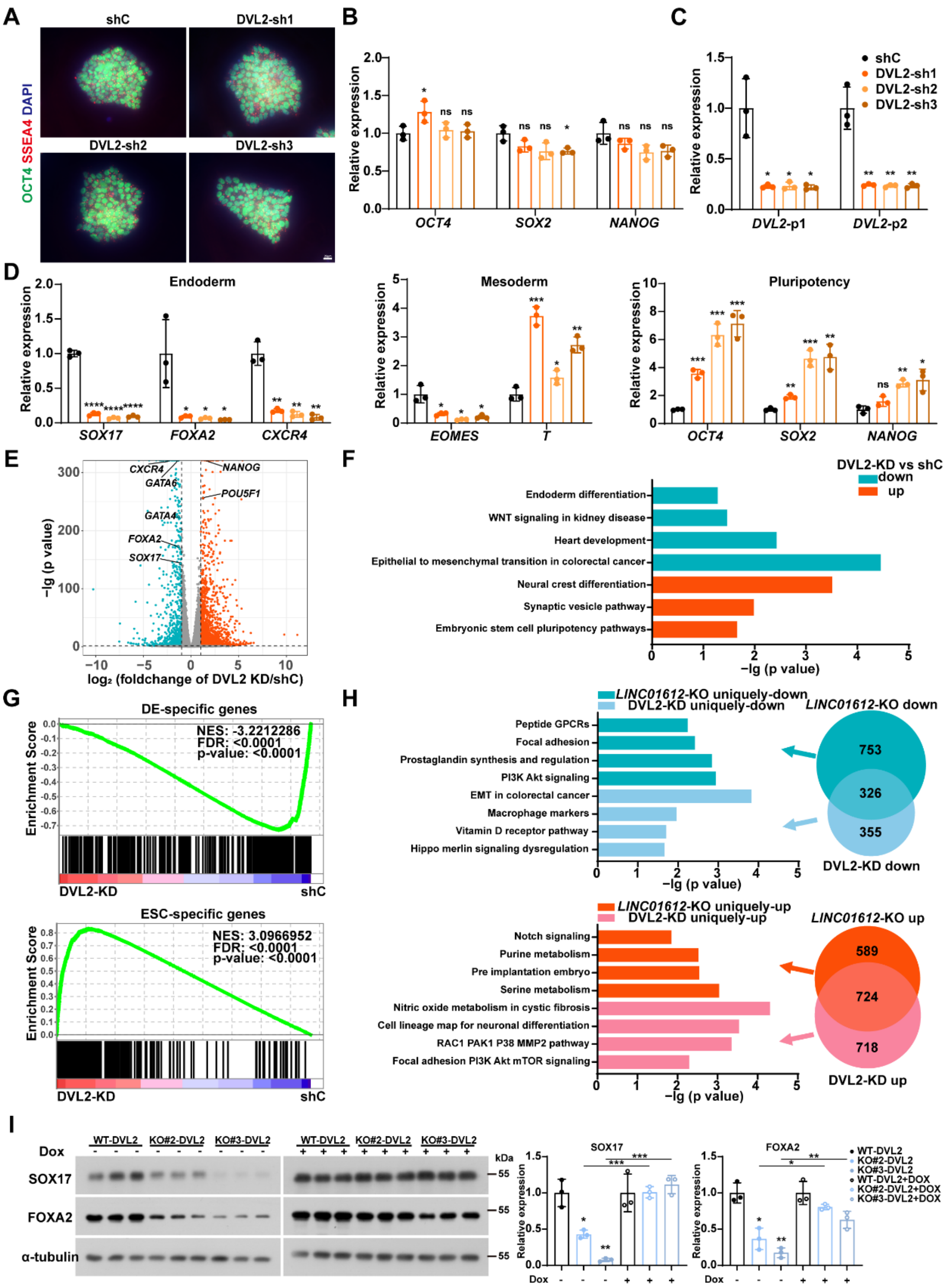

Figure S6. DVL2 functions in endoderm differentiation, related to Fig 5.

(A) Immunofluorescent staining of pluripotency markers (OCT4 and SSEA4) in shRNA control (shC) and DVL2-KD ESCs. Scale bar, 20 µm.

(B) The RNA levels of pluripotency genes (*OCT4*, *SOX2* and *NANOG*) in DVL2-KD and control ESCs (n = 3 independent experiments).

(C) Knockdown efficiencies of three *DVL2*-targeting shRNAs, determined by RT-qPCR in ESCs (n = 3 independent experiments).

(D) The RNA levels of endoderm genes (*SOX17*, *FOXA2* and *CXCR4*), mesoderm genes (*EOMES* and

T), and pluripotency genes (*OCT4*, *SOX2* and *OCT4*), in DVL2-KD and control DE cells (n = 3 independent experiments).

(E) Volcano plot showing differentially expressed genes identified by RNA-seq of control and DVL2-KD DE cells. Upregulated and downregulated genes upon DVL2-KD were shown in red and green, respectively.

(F) GO enrichment analysis of upregulated and downregulated genes in DVL2-KD DE cells, respectively.

(G) GSEA profile of DE-specific genes and ESC-specific genes in DVL2-KD and control DE cells.

(H) GO enrichment analysis of uniquely differentially expressed genes in *LINC01612*-KO and DVL2-KD DE cells.

(I) Western blot analysis showing the protein levels of SOX17 and FOXA2 in differentiated wildtype, *LINC01612*-KO and DVL2-overexpressing cells with *LINC01612*-KO. Quantitative results were shown at the bottom (n = 3 independent experiments).

Table S1. Sequences of two isoforms of *LINC01612*.

***LINC01612* isoform 1 sequences (5' to 3'): 1256bp**	GTGCTCTTGTGATTGTGACTGACACAGGCAGCACCCTTTCGCAGAAGT<br>AGATGTGCGTTGCTGAGAAATTTTCTGCCTAAGTTCTGGTTTTCTTTGC<br>AGCACCCAGCATTTGTTTCTAACACAGGGAAAGAGTGAGCTGTAAAAA<br>CCTCTCAAATTCAAGAAAGCGTAAAGCAAATTGAACAATACGGGTGAG<br>GAGCACGGACTATTCTCTCATGTTGAATGTGGCACTTTTTACATACAAG<br>CAGGAGAGCAACTACACCAAGATTTTGGAAAACATGTTATGTAAGGTT<br>AATTGTGACCTCTATGATGGGGATATGATAAAGAAGAAAAGTACCTCAC<br>AGCGATGATTGGCATGTGATCACTTAGCCAATGAGATAAGAGGCTGTAG<br>AAACGATGTGATAATGACATCTCCTGAGTTGCAGGTCCATATTAGAAGT<br>GGATATGAAGTAAACATTGAGCTTTCCTCACATTTAATTAACAAGCTTTA<br>TTATAGATACAGCTCAAATTAGTTAATTCAGGAAGCTTATTTTGTAGAAA<br>TTAAAACATTAATAAACACATACATTTAGAAATAAAGAACTCGGCTGAC<br>CCTTTGACTCTTTGAAACAAGTGGCCTTCCATGCTGTGAAAGATTGTAG<br>CCACTCTTTGAGACATTGGAATGGTGTCCATTGCATTTTATCAAGTTAAC<br>TATAGGTCAGCCTGGGAAATCTGAAGTTTCCTTATCTCCTGTCACTGTG<br>GTTGCTGTGAGATTTTGTGTCATCACTGTGCTGTCTTCCGTCTCCACATG<br>AAGGTGAGCTGTCTGGCTTCACTCTAACGTTTGTGATTTCACGCCCGAC<br>CTGATTGTTGCCAAGCAATTCCTTTGTTTGGAGAATTCTGTGCTTTGTTT<br>AGAATTTTTTAAAGGTTCTTTTCCTGTGGTCAGATTTCTGTTTGCTACTG<br>TGTCTTATGCCTTGTTCTCCTACAGAGAAGATTCTTTGAAGTTAGTGGA<br>AGCTCAGCTTAAAGGAAGATCTACTGCTCTGTGTGGTGGATGACCATG<br>GGGGAGCGAGAAGGCAGCCTCCGGCTTTTGTTTTGTGTGTGTGTGTGT<br>GTGTGTGTGTGATGTACCTGGAAAAATAACACGATTAGGATTAAAGAGT<br>ATGTTTGGCATGTTTAAAATAAATTGTAAACCAAACGTATTTTAAAGAAT<br>TTATACAAATTTGTCATTTAGTAAAATGAATGACTATTGAGTATTCATGTC<br>AATAAAACAACCACTTGAATACAACAAA
***LINC01612* isoform 2 sequences (5' to 3'): 1085bp**	GTGCTCTTGTGATTGTGACTGACACAGGCAGCACCCTTTCGCAGAAGT<br>AGATGTGCGTTGCTGAGAAATTTTCTGCCTAAGTTCTGGTTTTCTTTGC<br>AGCACCCAGCATTTGTTTCTAACACAGGGAAAGAGTGAGCTGTAAAAA<br>CCTCTCAAATTCAAGAAAGCGTAAAGCAAATTGAACAATACGGGTGAG<br>GAGCACGGACTATTCTCTCATGTTGAATGTGGCACTTTTTACATACAAG<br>CAGGAGAGCAACTACACCAAGATTTTGGAAAACATGTTATGTAAGGTT<br>AATTGTGACCTCTATGATGGGGATATGATAAAGAAGAAAAGTACCTCAC<br>AGCGATGATTGGCATGTGATCACTTAGCCAATGAGATAAGAGGCTGTAG<br>AAACGATGTGATAATGACATCTCCTGAGTTGCAGGTCCATATTAGAAGT<br>GGATATGAAGTAAACATTGAGCTTTCCTCACATTTAATTAACAAGCTTTA<br>TTATAGATACAGCTCAAATTAGTTAATTCAGGAAGCTTATTTTGTAGAAA<br>TTAAAACATTAATAAACACATACATTTAGAAATAAAGAACTCGGCTGAC<br>CCTTTGACTCTTTGAAACAAGTGGCCTTCCATGCTGTGAAAGATTGTAG<br>CCACTCTTTGAGACATTGGAATGGTGTCCATTGCATTTTATCAAGTTAAC<br>TATAGGTCAGCCTGGGAAATCTGAAGTTTCCTTATCTCCTGTCACTGTG<br>GTTGCTGTGAGATTTTGTGTCATCACTGTGCTGTCTTCCGTCTCCACATG<br>AAGAGAAGATTCTTTGAAGTTAGTGGAAGCTCAGCTTAAAGGAAGATC

	TACTGCTCTGTGTGGTGGATGACCATGGGGGAGCGAGAAGGCAGCCTCCGGCTTTTGTTTTGTGTGTGTGTGTGTGTGTGTGTGATGTACCTGGAAAAATAACACGATTAGGATTAAAGAGTATGTTTGGCATGTTTAAAATAAATTGTAAACCAAACGTATTTTAAAGAATTTATACAAATTTGTCATTTAGTAAAATGAATGACTATTGAGTATTCATGTCAATAAAACAACCACTTGAATACAACAAA

Table S2: The TPM of gene expression in wildtype, *LINC01612*-KO, shC and DVL2-KD DE cells. Provided in .xlsx file.

Table S3: The GO terms of the *LINC01612*-KO and DVL2-KD DEGs. Provided in .xlsx file.

Table S4: The mass spectrometry results of *LINC01612* or Fluc IP extracts. Provided in .xlsx file.

Table S5: Oligos used in this study.

Gene specific primers used in RACE assays and full-length cloning	
5-RACE-GSP1	GATTACGCCAAGCTTGTTTCAAAGAGTCAAAGGGTCAGCCGAG
3-RACE-GSP2	GATTACGCCAAGCTTGCAGAAGTAGATGTGCGTTGCTGAGAA
LINC01612-CDS-1256-F	GTGCTCTTGTGATTGTGACTGACA
LINC01612-CDS-1256-R	TTTGTTGTATTCAAGTGGTTGTTTTATTG
LINC01612-P1/2-F	GCACGGACTATTCTCTCATGTT
LINC01612-P1-R	AGGTTCTTTTCCTGTGGTCAGA
LINC01612-P2-R	CATGCCAAACATACTCTTTAATCCT
DVL2-CDS-2211-F	ATGGCGGGTAGCAGCACTGGGGGCGGT
DVL2-CDS-2211-R	CTACATAACATCCACAAAGAACTCGC
Fluc-F	AAGATGCCAAAAACATTAAGAAGG
Fluc-R	CACGGCGATCTTGCCGCCCTTCTT
LINC01612-1339-F	AACTCTGGGGACTCACAACACT
LINC01612-1339-R	CACTTACCTGGAATAAAGACTTGGG
Target gene	shRNA target sequence
LINC01612-sh1	GCAGGTCCATATTAGAAGT
LINC01612-sh2	GATTGGCATGTGATCACTT
Control (shC)	GAAGTATTCCGCGTACGTT
DVL2-sh1	GGGTGGTGAAGGAAGAAATTT
DVL2-sh2	AGCGTCACAGATTCCACAATG
DVL2-sh3	GCCCACTTTCTCCTACCAATA
Target gene	sgRNAs target sequence
LINC01612-KO-g1	ATTATGTATCCAAATCATCG
LINC01612-KO-g2	AAGCTAAGATGAGTAGGCTA
LINC01612-ORF1-K1-g1	ACATGCCAATCATCGCTGTG
LINC01612-ORF3-KI-g1	AAGTACCTCACAGCGATGAT
Gene name	qPCR primers
NANOG	F: GAGACTTGGCACGCCTGT
	R: CCAGGTTGAATTGTTCCAGGTC
OCT4	F: CCCCAGCCTTTACTCTTCCTA
	R: CCAGGTTGAATTGTTCCAGGTC

SOX2	F: CAAAGCAGAAACCCTCGTGC
	R: TCTCACTCGGTTCTCGATACTG
MIXL1	F: GTCATTTGCTGTGGGTGATG
	R: AGAAAAACGAGGGAAATGGG
EOMES	F: CACATTGTAGTGGGCAGTGG
	R: CGCCACCAAACTGAGATGAT
T	F: GAGACTTGGCACGCCTGT
	R: GGTACCCCGACATCCACTT
SOX17	F: GATGATCGTGACCAAGAACGG
	R: CCACGAAGTCCAGCAGGAA
FOXA2	F: GCATGACTCCGGTGTGAATCT
	R: TCACACGTCAGGATAGTTGCAGT
GATA4	F: GGAGCAGCTACTATGCAGAGC
	R: CGTGTTCATGCCGTTCATCC
GATA6	F: CAGGCGTTGCACAGATAGTG
	R: CCCGACACCCCAATCTC
CXCR4	F: AGTTCCTACGCTTCGCATCCCTTC
	R: TGAACAGCAGCAAGTCCTCCCA
GAPDH	F: TACACCGAGGAAATGGGCTCA
	R: AGATGATGGAGTAGATGGTGGG
MALAT1	F: AATGAAGGGGTCATTGATGG
	R: AAGGTGAAGGTCGGAGTCAA
LINC01612-p1	F: GCAGGAGAGCAACTACACCAA
	R: GCTAAGTGATCACATGCCAATC
LINC01612-p2	F: CTGTGCTGTCTTCCGTCTCC
	R: TCGGGCGTGAAATCACAAAC
LINC01612-p3	F: AGGGAAAGAGTGAGCTGTAAAAAC
	R: TCACCCGTATTGTTCAATTTGCTTT
DVL2-p1	F: TCAGCAGCGTCACAGATTCC
	R: GTCTCCCCGCTCATTGCTC
DVL2-p2	F: GAGGAAGAGACTCCCTACCTG
	R: CGGGCGTTGTCATCTGAAAT
PDX1	F: CCTTTCCCATGGATGAAGTC
	R: GAACTCCTTCTCCAGCTCTA
NKX6-1	F: AGACCCACTTTTTCCGGACA
	R: CCAACGAATAGGCCAAACGA
MEF2C	F: CTGGTGTAACACATCGACCTC
	R: GATTGCCATACCCGTTCCCT
ISL1	F: GCAGAGTGACATAGATCAGCCTG
	R: GCCTCAATAGGACTGGCTACCA
TNNT2	F: AAGAGGCAGACTGAGCGGGAAA
	R: AGATGCTCTGCCACAGCTCCTT

Supplemental Methods

Quantitative RT-qPCR and droplet digital PCR

Total RNA was extracted from cultured cells using the Hipure Total RNA Mini Kit (Magen, Cat#R4111-03) or TriPure Isolation Reagent (Roche, Cat#11667165001) following the manufacturer's protocols. cDNA was synthesized from 1 μg of total RNA using the ABScript II RT Master Mix (ABclonal, Cat#RK20402). Gene expression was quantified using 2×SYBR Green Fast qPCR Mix (ABclonal, Cat#RK21203) on a CFX384 qPCR system (Bio-Rad), with *GAPDH* as the internal control for normalization. The primers used in all RT-qPCR assays were listed in Table S5.

The copy numbers of *LINC01612* were quantified by ddPCR outsourced to Guangzhou Forevergen Biosciences. Briefly, the 20 μL PCR reaction mixture consisted of 1.8 μL forward primer, 1.8 μL reverse primer, 4.4 μL H_2O, 2 μL cDNA, and 10 μL ddPCR Supermix. Droplet generation was performed using MicroDrop-100A. 40 μL of the emulsion was then transferred into the 96-well PCR plate, which was then sealed with pierceable heat-seal film using a preheated heat sealer. The plate was cycled to an endpoint as per the manufacturer's protocol, then placed in the MicroDrop-100B biochip reader for detection and analyzed with QuantDrop software.

Flow cytometry

DE cells were dissociated with TrypLE (Gibco, Cat#12604021) and washed with DPBS containing 2% FBS. Cells were incubated with CD184-APC (BD, Cat#555976) for 30 minutes, fixed with Transcription Factor Buffer Set (BD, Cat#562574), and stained with SOX17-Alexa 488 (BD, Cat#562205). Isotype control was used. $SOX17^+$ or $CXCR4^+$ cells were analyzed using a flow cytometer (ACEA NovoCyte or CytoFLEX), with data analyzed by FlowJo software.

Immunofluorescence staining

Cells were fixed with 4% paraformaldehyde following PBS washes, then permeabilized and blocked using blocking buffer containing 10% donkey serum and 0.3% Triton X-100. Afterward, cells were incubated overnight at 4°C with primary antibodies at the appropriate dilution. The antibodies used included: SOX17 (R&D, Cat#AF1924, 1:200), FOXA2 (HuaBio, Cat#ET1703-76, 1:200), OCT4 (CST, Cat#2750, 1:200), SOX2 (BD, Cat#561469, 1:200), and SSEA4 (CST, Cat#4755, 1:200). After three PBS washes, the cells were incubated with secondary fluorescent antibodies. The cells were then incubated with DAPI solution for 5-10 minutes at room temperature, protected from light. Images were captured and analyzed using an Olympus IX53 microscope.

Western blot

Cell pellets were lysed in RIPA buffer (Beyotime, Cat#P0013C) with protease inhibitors (Roche, Cat#4693132001) for 30 minutes at 4°C. After centrifugation, supernatants were collected, and proteins were separated by 10% SDS-PAGE and transferred to nitrocellulose membranes (Millipore, Cat#Z746010). The membranes were washed with TBST, blocked with 5% skimmed milk in TBST for 30 minutes, and incubated with primary antibodies overnight at 4°C or for 2 hours at room temperature. After washing, the membranes were incubated with HRP-conjugated secondary antibodies for 1 hour. Signals were detected using ECL (Millipore, Cat#WBUSLS0100) and a film imaging system. Primary antibodies included: SOX17 (R&D, Cat#AF1924, 1:1000), FOXA2 (R&D, Cat#AF2400, 1:1000), GAPDH (Proteintech, Cat#10494-1-AP, 1:5000), α-Tubulin (Proteintech, Cat#11224-1-AP, 1:5000), active β-catenin (CST, Cat#8814, 1:1000), β-catenin (CST, Cat#8480, 1:1000), DVL2 (Proteintech, Cat#12037-1-AP, 1:1000), GFP (SANTA CRUZ, Cat#sc-101536, 1:1000) and Flag (Sigma, Cat# F1804, 1:1000).

RACE and cDNA cloning

After 3 days of DE differentiation, HUES8 cells were harvested, and total RNA was isolated using the Hipure Total RNA Mini Kit (Magen, Cat#R4111-03). The 5' and 3' regions of *LINC01612* were amplified with the SMARTer RACE 5'/3' kit (TAKARA, Cat#634858) according to the manufacturer's instructions. The resulting PCR products were then cloned and sequenced by Sanger sequencing. Gene-specific primers, along with those used to clone the full-length *LINC01612* transcripts, were provided in Table S5.

Cytosolic/nuclear fractionation

Approximately 1 million DE cells were collected by TrypLE and re-suspended in DPBS. Cells were lysed in CE buffer (10 mM Hepes, 60 mM KCl, 1 mM EDTA, 0.34 M sucrose, 0.3% NP-40, 1 mM DTT) with inhibitors on ice for 10 minutes, then centrifuged at 3000 rpm for 10 minutes to separate nuclear and cytoplasmic fractions. RNA was extracted using TriPure reagent (Roche, Cat#11667165001) and analyzed by RT-qPCR.

RNA pull-down

In brief, biotin-labeled *luciferase (Fluc)*, *LINC01612* and truncated *LINC01612* fragments were transcribed *in vitro* using HiScribe T7 High Yield RNA Synthesis Kit (NEB, Cat#E2040S) and biotin-16-UTP (Roche, Cat#11388908910) according to the manufacturer's instructions. A total of 10 μg of biotin-labeled *LINC01612* or truncated *LINC01612* were incubated with whole-cell lysates from DE cells or HEK293T cells. Subsequently, 40 μL of Dynabeads MyOne Streptavidin C1 (Invitrogen, Cat#65001) was used to capture RNA-protein complexes. These complexes were then analyzed by silver staining, mass spectrometry (provided by Institute of Hydrobiology, Chinese Academy of Science), and Western blot.

RNA immunoprecipitation (RIP)

RIP assays were carried out using the Magna RNA-binding protein immunoprecipitation kit (Millipore, Cat#17–700) according to the manufacturer's protocol. Cell lysates were incubated overnight at 4 °C with Protein A/G Magnetic Beads (MCE, Cat#HY-K0202) and the appropriate antibodies. RNA purification was achieved using proteinase K treatment, followed by extraction with TRIzol (Invitrogen, Cat#10296010) and phenol/chloroform/isoamyl alcohol. Quantitative analysis of the purified RNA was then performed using RT-qPCR.

Co-immunoprecipitation

The Co-IP experiment was mainly conducted based on the published experimental method (Ponzielli et al., 2013). Cells cultured on 10-cm dish were washed with PBS and lysed using IP WCE buffer (25 mM Tris pH 7.6, 0.5% NP-40, 250 mM NaCl, 3 mM EDTA, 3 mM EGTA, 1× protease inhibitor cocktail, 1 mM PMSF, and 0.5 mM DTT) on ice. After centrifugation to clarify the cell lysate, the supernatant was incubated with 2.5 μg of DVL2 or IgG antibody at 4 °C overnight with rotation, followed by an additional 3 hours' incubation with Protein A/G Magnetic Beads at 4 °C. The immunoprecipitates were washed with IP Wash Buffer (25 mM Tris pH 7.6, 1% NP-40, 200 mM NaCl, 3 mM EDTA, and 3 mM EGTA), then resuspended in 40 μL of 1× SDS loading buffer. The samples were analyzed by Western blot.

CHX, MG132 and BafA1 treatments

DE cells and HEK293T cells were treated with CHX (MCE, Cat#HY-12320) and collected at different time points. Similarly, cells were treated with MG132 (MCE, Cat#HY-13259) or BafA1 (MCE, Cat#HY-100558) followed by collection at 0- and 6-hour post-treatment. Proteins were subsequently extracted and analyzed by Western blot.

REFERENCES

Ponzielli, R., Tu, W.B., Jurisica, I., and Penn, L.Z. (2013). Identifying Myc interactors. Methods Mol Biol *1012*, 51-64. 10.1007/978-1-62703-429-6_4.
