# Supplementary material for: The LINC01612-DVL2-WNT axis promotes human endoderm differentiation
Source: Stem Cell Reports. 2025 Oct 23;20(11):102682. doi: 10.1016/j.stemcr.2025.102682 (PMC12790727; doi:10.1016/j.stemcr.2025.102682)
Supplement: Document S2. Article plus supplemental information [file mmc5.pdf]

Stem Cell Reports
Article
OPEN ACCESS

The *LINC01612*-DVL2-WNT axis promotes human endoderm differentiation

Mao Li,[1,5] Pei Lu,[1,5] Jie Yang,[1,2,5] Chenchao Yan,[1] Yikang Yang,[1] and Wei Jiang[1,3,4,6,*]
[1]Department of Biological Repositories, Frontier Science Center for Immunology and Metabolism, Medical Research Institute, Zhongnan Hospital of Wuhan University, Wuhan University, Wuhan 430071, China
[2]State Key Laboratory of Biocatalysis and Enzyme Engineering, School of Life Sciences, Hubei University, Wuhan 430062, China
[3]Human Genetics Resource Preservation Center of Wuhan University, Wuhan 430071, China
[4]Hubei Provincial Key Laboratory of Developmentally Originated Disease, Wuhan 430071, China
[5]These authors contributed equally
[6]Lead contact
*Correspondence: jiangw.mri@whu.edu.cn
https://doi.org/10.1016/j.stemcr.2025.102682

SUMMARY

Long noncoding RNAs in gene desert regions remain largely uncharacterized despite their potential regulatory roles in cell differentiation. Here, we identify *LINC01612* as a crucial modulator of human definitive endoderm differentiation. *LINC01612* exhibits stage-specific expression and lacks protein-coding potential during endoderm differentiation. Depletion of *LINC01612*, through either short hairpin RNA (shRNA)-mediated knockdown or promoter deletion, severely impairs human endoderm differentiation. Mechanistically, *LINC01612* interacts with DVL2, a WNT regulator essential for early development, and enhances DVL2 protein stability by reducing its ubiquitination. Loss of *LINC01612* or DVL2 impairs WNT signaling, while both WNT activation and DVL2 overexpression can rescue endoderm differentiation defect in the absence of *LINC01612*. These findings reveal the *LINC01612*-DVL2-WNT regulatory axis as a key modulator of human definitive endoderm differentiation.

INTRODUCTION

Long noncoding RNAs (lncRNAs) are a diverse class of RNA molecules that are longer than 200 nucleotides without protein-coding potential, playing crucial roles in gene regulation (Chen and Kim, 2024). Over the past decade, accumulating evidence has revealed that lncRNAs participate in a wide range of biological processes, including chromatin remodeling, transcriptional regulation, post-transcriptional modifications, and signal transduction (Lu et al., 2021). LncRNAs regulate gene expression in a context-dependent manner by interacting with DNA, RNA, or proteins to act as scaffolds, decoys, or guides (Mattick et al., 2023; Wang and Chang, 2011). In general, *cis*-acting lncRNAs, which constitute a substantial portion of functionally characterized lncRNAs, regulate nearby gene expression across varying genomic distances (Gil and Ulitsky, 2020; Quinn and Chang, 2016). In contrast, *trans*-acting lncRNAs, particularly those located distally from protein-coding genes (PCGs) and named as "desert lncRNAs (far away from PCGs more than 50 kb in the genome)" (Lu et al., 2023), are transcribed and translocated from their sites of origin to regulate gene expression at distant genomic loci (Kopp and Mendell, 2018; Statello et al., 2021). Our early studies showed that desert lncRNAs exhibit high expression levels specific to certain cell types or developmental stages compared to those lncRNAs near PCGs (Lu et al., 2023; Yang et al., 2025). These findings highlight potential stage-specific functions of desert lncRNAs mediated by distinct regulatory mechanisms. However, the study of desert lncRNAs remains limited, primarily because of the difficulties in uncovering their biological roles and pinpointing their downstream targets.

Embryonic stem cells (ESCs), which originate from the inner cell mass (ICM) of the blastocyst, can self-renew and differentiate into the three germ layers: ectoderm, mesoderm, and definitive endoderm (DE) (Thomson et al., 1998; Zhai et al., 2022). Due to the ethnic and technical limitations to access human materials, human ESCs offer a valuable model for studying early development, disease modeling, drug screening, and cell therapy (Yiangou et al., 2018). During embryonic patterning and the establishment of germ layers, a combination of elevated Activin or Nodal signaling with moderate WNT signaling collaboratively triggers the DE differentiation (D'Amour et al., 2005; Loh et al., 2014). Activating both signaling pathways further results in the expression of key endodermal transcription factors, such as *SOX17*, *FOXA2,* and *GATA4/6* (Ang et al., 1993; Heslop et al., 2021; Viotti et al., 2014). The DE further gives rise to the respiratory and gastrointestinal systems, along with the derivate tissues and organs (Zorn and Wells, 2009), holding great significance for regenerative medicine and disease studies.

Accumulating evidence underscores the significance of lncRNAs as essential regulators during embryonic development and cell differentiation. LncRNAs control the lineage specification and differentiation potential of ESCs, guiding processes such as neurogenesis, myogenesis, and cardiogenesis and the differentiation into endodermal lineages (Flynn and Chang, 2014; Lu et al., 2021; Mirzadeh Azad et al., 2021; Yan et al., 2017). For instance, lncRNAs *DEANR1* and *GATA6-AS1* are vital for endoderm

Published by Elsevier Inc. on behalf of International Society for Stem Cell Research.
This is an open access article under the CC BY license (http://creativecommons.org/licenses/by/4.0/).

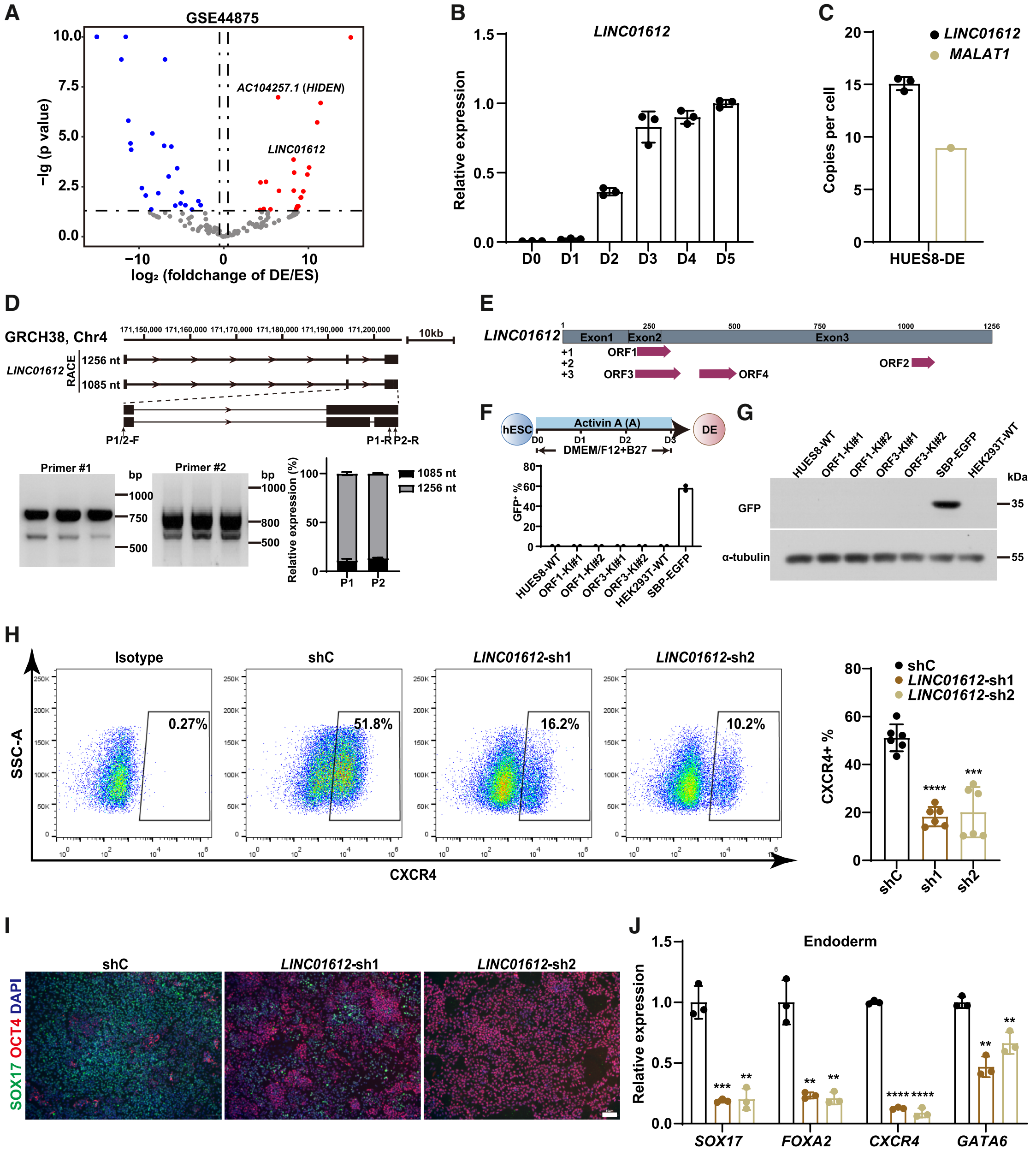

Figure 1. *LINC01612* is a noncoding RNA highly expressed in human endoderm lineage

(A) Scatterplot of differentially expressed desert lncRNAs between ESCs and DE cells. Red indicates higher expression, while blue indicates lower expression.

(B) Time-course expression of *LINC01612* during human endoderm differentiation, detected by RT-qPCR (*n* = 3 independent experiments).

(C) The copy number per cell of *LINC01612* in HUES8-derived DE cells, detected by droplet digital PCR. *MALAT1* was used as a control.

(D) Diagram of the *LINC01612* gene locus. Two isoforms of *LINC01612* were identified by 5′ and 3′ RACE and validated by RT-PCR. The relative expression levels of the two isoforms were calculated according to PCR analysis (*n* = 3 independent experiments).

(E) The protein-coding potential of *LINC01612* was predicted using ORF Finder online tool.

(F and G) Top panel: schematic representation of endoderm differentiation of human ESCs. Bottom panel: Flow cytometric analysis (F) and western blot (G) showing the expression of ORF-EGFP (*n* = 2 independent experiments) in ORF1/3 knockin HUES8-DE cells. SBP-EGFP (overexpressing EGFP in HEK293T cells) was used as a positive control to confirm the expression of GFP vector.

(H) Flow cytometric analysis of CXCR4-positive cells in shRNA control (shC) and *LINC01612*-KD DE cells. The statistical results were shown on the right (*n* = 3 independent experiments).

(legend continued on next page)

differentiation by promoting the expression of nearby DE genes *FOXA2* and *GATA6,* respectively, by recruiting SMAD2/3 to their promoter regions (Jiang et al., 2015; Yang et al., 2020). The lncRNA *DIGIT* could regulate endoderm differentiation by modulating GSC transcription (Daneshvar et al., 2016); moreover, *DIGIT* interacts with BRD3 to promote phase separation, facilitating BRD3 binding to H3K18ac-enriched regions of endodermal transcription factors (Daneshvar et al., 2020). A notable observation is that these endoderm-associated lncRNAs identified so far are located near PCGs and exert regulatory effects on the transcription of neighboring endoderm-related genes, thereby playing a role in endoderm differentiation. Our group recently demonstrated that a desert lncRNA *HIDEN* enhances the interaction between IMP1 protein and *FZD5* mRNA, promoting human DE differentiation (Lu et al., 2023). However, many other desert lncRNAs are still awaiting functional dissection.

This study identifies *LINC01612* as a new DE-specific desert lncRNA highly expressed during human DE differentiation. Its depletion significantly impairs human endoderm differentiation by downregulating WNT activity. We further dissect the underlying mechanism and reveal that *LINC01612* interacts with and stabilizes DVL2 by reducing its ubiquitination, thereby promoting WNT signaling. These findings reveal that the *LINC01612*-DVL2-WNT axis is essential for human DE differentiation.

RESULTS

LINC01612 is a lncRNA highly expressed in human endoderm lineage

To investigate the role of desert lncRNAs (more than 50 kb far away from PCGs in the genome) during human early differentiation, we conducted transcriptome analysis on our published data (GEO: GSE44875) using ESCs and sorted CD184/CD117-double positive DE cells (Jiang et al., 2015), identifying 46 differentially expressed desert lncRNAs (Lu et al., 2023). Notably, *LINC01612* exhibited significantly elevated expression in DE cells (Figures 1A and S1A). A time-course analysis revealed a gradual upregulation of *LINC01612* during DE differentiation (Figure 1B). Furthermore, analysis of *LINC01612* expression in 30 human tissues from the Genotype-Tissue Expression (GTEx) database demonstrated its high expression in endoderm-derived tissues such as the liver and lung (Figure S1B). To assess the accurate expression level of *LINC01612* at the single-cell level, we performed droplet digital PCR, and the result revealed that in HUES8-DE cells, the copy number of *LINC01612* is comparable to that of lncRNA *MALAT1* (Figure 1C), a key lncRNA that is highly expressed in various cancers and during development and mainly localizes to nuclear speckles to regulate cell proliferation via mechanisms involving alternative splicing and transcriptional control (Amodio et al., 2018). Given the imprecise annotation of lncRNAs (St Laurent et al., 2015), we conducted 5′ and 3′ rapid amplification of cDNA ends (RACE) experiments, revealing two isoforms of *LINC01612* differing by a single region: one was 1,256 nucleotides and the other was 1,085 nucleotides (Figure 1D and Table S1). Next, we further assessed the expression levels of both isoforms and found that the longer isoform accounts for more than 85% (Figure 1D). Therefore, we later concentrated on the longer isoform for our subsequent studies.

Recently, an increasing number of lncRNAs containing small open reading frames (smORFs) have been reported to translate into functional micropeptides (Andrews and Rothnagel, 2014; Pang et al., 2018). To test such a possibility, we used ORF Finder (https://www.ncbi.nlm.nih.gov/orffinder/) to predict whether *LINC01612* could encode a micropeptide. We identified four potential smORFs (Figures 1E and S1C). To examine the coding potential of *LINC01612*, we fused EGFP (lacking the start codon ATG) in frame to the C terminus of these four smORFs within the *LINC01612* transcript, immediately upstream of their stop codons (Figure S1C). After transfecting these plasmids into HEK293T cells, we observed the expression of ORF1-EGFP and ORF3-EGFP fusion proteins (Figures S1D–S1F), similar to the reported coding micropeptide as a positive control (D’Lima et al., 2017). Moreover, when the EGFP sequence (without the start codon ATG) was fused to the C terminus of ORF1 and ORF3 following their stop codons, it abolished the expression of the ORF1-EGFP and ORF3-EGFP fusion proteins (Figures S1D and S1F), indicating that ORF1 and ORF3 likely encode micropeptides in HEK293T cells. We further performed CRISPR-Cas9-mediated *in situ* knockin of ORF-EGFP in HUES8 ESCs to verify the endogenous translational activity of ORF1 and ORF3 and finally generated four EGFP knockin clones (two clones for each ORF). Then, wild-type and these ORF-EGFP knockin ESCs were subjected to DE differentiation induced by Activin signaling (Figure 1F). However, we failed to

(I) Immunofluorescent staining of SOX17 and OCT4 in shC and *LINC01612*-KD DE cells. Scale bar, 50 μm.
(J) The RNA levels of endoderm genes (*SOX17*, *FOXA2*, *CXCR4*, and *GATA6*) in *LINC01612*-KD and control DE cells (n = 3 independent experiments).
Data are presented as the mean ± SD. Significance levels are indicated as $^{**}p < 0.01$, $^{***}p < 0.001$ and $^{****}p < 0.0001$.

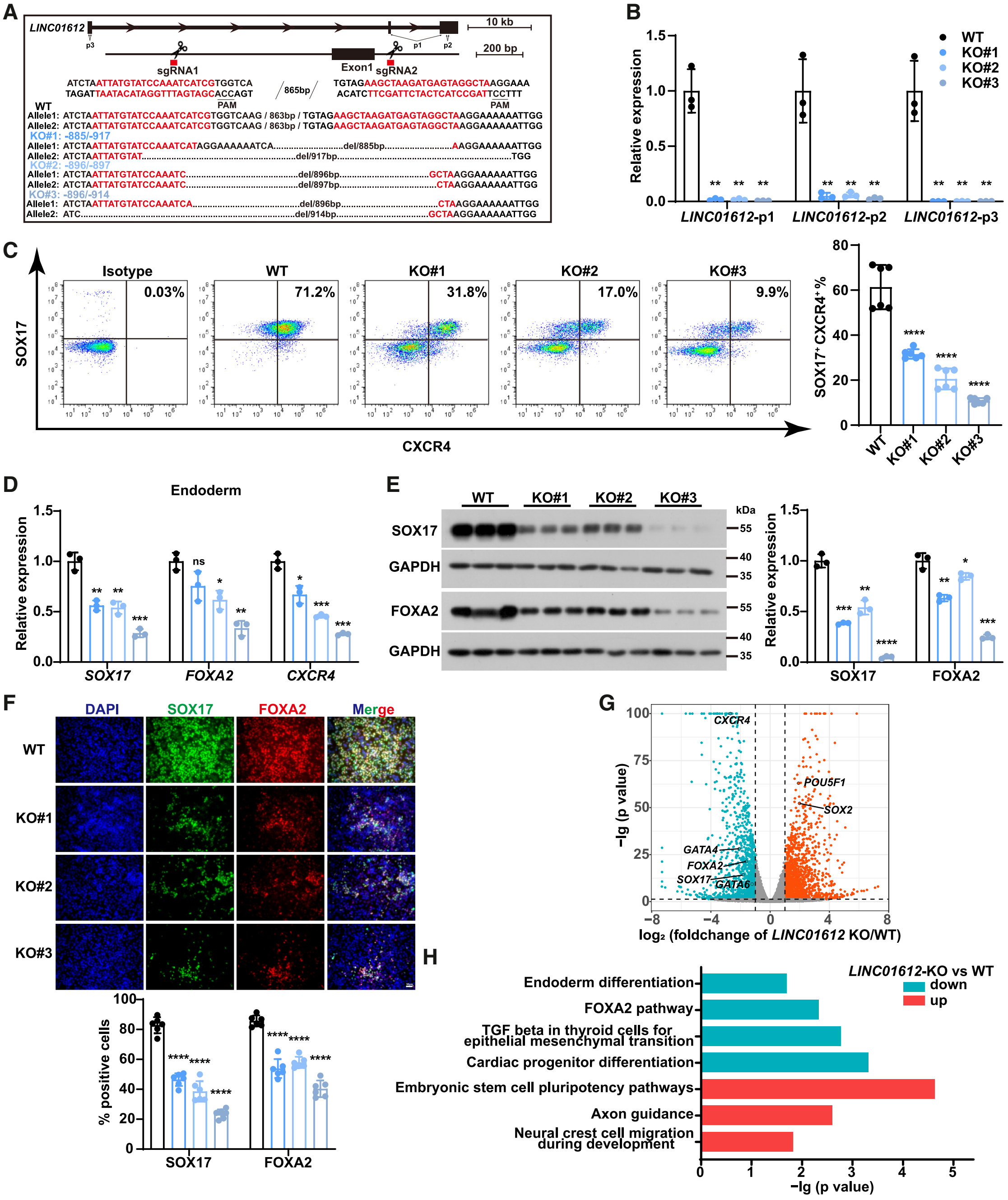

Figure 2. Knockout of *LINC01612* severely affects DE differentiation

(A) The genotypes of the three *LINC01612*-KO HUES8 ESC lines. The sgRNA target sequences and PAM sequences were indicated in red and underlined, respectively.

(B) The *LINC01612* expression levels in wild-type and *LINC01612*-KO DE cells, examined by RT-qPCR using three sets of primers illustrated in (A) ($n = 3$ independent experiments).

(C) Flow cytometric analysis of SOX17- and CXCR4-positive cells in wild-type and *LINC01612*-KO DE cells. The statistical results of double-positive cells were shown on the right ($n = 6$ independent experiments).

(D) The RNA levels of endoderm genes (*SOX17*, *FOXA2* and *CXCR4*) in wild-type and *LINC01612*-KO DE cells ($n = 3$ independent experiments).

(E) The protein levels of DE markers (SOX17 and FOXA2) in wild-type and *LINC01612*-KO DE cells, determined by western blot. Quantitative results were shown on the right ($n = 3$ independent experiments).

(legend continued on next page)

observe any ORF-EGFP signal by flow cytometry analysis or western blot, although the differentiation was successfully validated by CXCR4 (CD184)-based flow cytometric analysis (Figures 1F, 1G, and S1G). Taken together, these findings indicate that *LINC01612* is highly expressed but less likely encodes protein during DE differentiation, despite the fact that we could not exclude the coding potential in other contexts.

To explore the function of *LINC01612* in DE differentiation, we established two stable *LINC01612*-knockdown (KD) ESC lines using shRNAs, achieving at least 85% knockdown efficiency as determined by RT-qPCR (Figures S1H and S1I). No significant differences in pluripotent markers, such as OCT4 (POU5F1) and SSEA4, were observed between control and *LINC01612*-KD ESCs (Figure S1H). Importantly, we found that *LINC01612*-KD cells exhibited lower DE differentiation efficiency, evident by CXCR4-based flow cytometric analysis (Figure 1H) and further supported by the expression of SOX17 using immunofluorescence analysis (Figure 1I). RT-qPCR assay showed that the RNA levels of endoderm genes (*SOX17*, *FOXA2*, *CXCR4,* and *GATA6*) were significantly reduced (Figure 1J) and the expression of pluripotent genes (*SOX2* and *OCT4*) and mesodermal genes (*MIXL1* and *T*) were increased in *LINC01612*-KO DE cells compared with control (Figure S1J). Taken together, these data indicate that *LINC01612* KD severely impairs human ESC differentiation toward DE.

LINC01612 is required for endoderm differentiation

Furthermore, we employed CRISPR-Cas9-mediated genomic deletion to completely abolish *LINC01612* function. Based on the observed signal enrichment of assay for transposase-accessible chromatin using sequencing (ATAC-seq) (GEO: GSE285132) indicating chromatin accessibility and H3K27ac chromatin immunoprecipitation sequencing (ChIP-seq) peaks (data from ENCODE: https://www.encodeproject.org/experiments/ENCSR200ETW/) marking transcriptionally active regions (Figure S2A), we defined the genomic region adjacent to exon 1 of *LINC01612* as its promoter and accordingly designed two single guide RNAs (sgRNAs) to delete this region to achieve complete ablation (Figures 2A and S2A). Genomic PCR and RT-qPCR results validated successful knockout and complete deletion of *LINC01612* transcript in three *LINC01612*-KO clones with different genotypes (Figures 2A, 2B, and S2B). We found that the mRNA and protein expression levels of pluripotency-associated genes were not significantly altered upon *LINC01612*-KO (Figures S2C and S2D). Next, we subjected the *LINC01612*-KO and wild-type ESCs to DE differentiation. Flow cytometric analysis showed that DE differentiation was compromised (Figure 2C), and the expression of key endodermal genes, including *SOX17*, *FOXA2*, and *CXCR4*, was significantly reduced upon *LINC01612*-KO, whereas pluripotency- and mesoderm-associated genes were upregulated (Figures 2D and S2E). Similarly, western blot analysis and immunofluorescence staining revealed a significant reduction of SOX17 and FOXA2 protein levels in *LINC01612*-KO DE cells (Figures 2E and 2F). Taken together, the loss of *LINC01612* results in a pronounced impairment in DE differentiation of human ESCs.

Next, we performed RNA sequencing (RNA-seq) to evaluate the transcriptomic change upon *LINC01612* deletion during DE differentiation (Table S2). We identified 1,079 downregulated genes and 1,313 upregulated genes, and consistent with RT-qPCR results, the DE marker genes (*SOX17*, *FOXA2*, *CXCR4*, and *GATA4/6*) were downregulated, while the pluripotent genes (*POU5F1* and *SOX2*) were upregulated (Figure 2G). In addition, gene ontology (GO) analysis revealed that the terms for downregulated genes in *LINC01612*-KO DE cells enriched in endoderm differentiation, FOXA2 pathway, and transforming growth factor β (TGF-β) for EMT. In contrast, the upregulated genes were related to ESC pluripotency pathways, axon guidance, and neural crest cell migration during development (Figure 2H and Table S3). Gene set enrichment analysis (GSEA) also showed a significant downregulation of DE-specific genes and an upregulation of ESC-specific genes in *LINC01612*-KO DE cells (Figure S2F).

To verify the important role of *LINC01612* in lineage differentiation, we performed embryoid body (EB) differentiation and analyzed gene expression by RT-qPCR (Figures S3A and S3B). Compared to wild-type cells, *LINC01612*-KO cells showed reduced expression of endoderm markers, while ectoderm markers were obviously upregulated (Figure S3B), supporting the crucial role of *LINC01612* in the development of endoderm lineages. However, the expression of mesoderm markers was somehow chaotic: *T* was significantly upregulated, while *MIXL1* was downregulated to a certain extent (Figure S3B). To further investigate the role of *LINC01612* during the progressive

(F) Immunofluorescent staining of DE markers (SOX17 and FOXA2) in wild-type and *LINC01612*-KO DE cells. Quantitative results were shown at the bottom (n = 6 images). Scale bar, 50 μm.
(G) Volcano plot showing differentially expressed genes identified by RNA-seq of wild-type and *LINC01612*-KO DE cells. Upregulated and downregulated genes upon *LINC01612*-KO were shown in red and green, respectively.
(H) GO enrichment analysis of upregulated and downregulated genes in *LINC01612*-KO DE cells compared to wild-type, respectively.
Data are presented as the mean ± SD. Significance levels are indicated as *p < 0.05, **p < 0.01, ***p < 0.001 and ****p < 0.0001; ns, not significant.

differentiation of mesendodermal cells into more specific lineages, we further induced endodermal pancreatic differentiation (Li et al., 2024) and mesodermal heart progenitor differentiation (Yang et al., 2024) in wild-type and *LINC01612*-KO cells. We observed significant downregulation of pancreatic transcription factors in *LINC01612*-KO cells, including PDX1 at pancreatic progenitor (PP) 1 stage and PDX1 and NKX6-1 at PP2 stage (Figures S3C and S3D). Similarly, the expression of heart progenitor markers (*MEF2C*, *ISL1*, *GATA4,* and *TNNT2*) was reduced as well (Figure S3E). Collectively, these results underscore the essential function of *LINC01612* in human mesendodermal lineage differentiation.

WNT signaling pathway acts as the downstream of *LINC01612*

To understand how *LINC01612* affected human endoderm differentiation, we performed GO analysis of differentially expressed genes in *LINC01612*-KO cells, showing enrichment in pathways related to pluripotent stem cell differentiation pathway, WNT signaling, and TGF-β receptor signaling (Figure 3A and Table S3). Given the critical role of the WNT signaling pathway in endoderm differentiation (Dziedzicka et al., 2021; Jiang et al., 2013), we hypothesized that the WNT signaling pathway might function downstream of *LINC01612*. Therefore, we analyzed the protein levels of active (unphosphorylated, nuclear-located) β-catenin, the key effector of the WNT signaling pathway, in wild-type and *LINC01612*-KO DE cells. Both active and total β-catenin levels were significantly diminished in *LINC01612*-KO DE cells compared to wild-type cells (Figure 3B). We also observed a significant increase in both active and total β-catenin protein levels in *LINC01612*-overexpressing HEK293T cells (Figures S4A and S4B). Additionally, the β-catenin/TCF-responsive luciferase reporter assay in HEK293T cells revealed that *LINC01612* overexpression resulted in significantly increased TCF luciferase activity (Figure 3C). These findings suggest that *LINC01612* depletion, indeed, impairs WNT signaling pathway during DE differentiation.

WNT signal activation is essential for efficient DE differentiation, particularly during the early mesendoderm differentiation phase (Jiang et al., 2013; Loh et al., 2014). We wondered whether the activation of WNT signalg could rescue the impaired DE differentiation observed in *LINC01612*-KO cells. Therefore, we treated *LINC01612*-KO cells with either GSK3 inhibitor CHIR-99021 or WNT ligand protein Wnt3a (both function as WNT signal activators) (Figure 3D). RT-qPCR analysis revealed a significant restoration of DE-associated genes (*SOX17* and *FOXA2*) in *LINC01612*-KO DE cells treated with CHIR-99021 or Wnt3a (Figure 3D). Consistently, flow cytometry and western blot analyses also showed significant restoration of SOX17 and FOXA2 expression in *LINC01612*-KO DE cells treated with CHIR-99021 or Wnt3a (Figures 3E–3G and S4C). These results suggest that modulating the WNT signaling pathway can resolve the DE differentiation defects induced by *LINC01612* disruption. This supports the notion that WNT signaling is the functional downstream of *LINC01612*, critical in regulating DE differentiation.

LINC01612 physically interacts with and stabilizes DVL2 protein by reducing ubiquitination

To investigate how *LINC01612* affects WNT activity and contributes to DE differentiation, we first assessed its subcellular localization in DE cells. We separated the cytoplasmic and nuclear fractions of DE cells and found that the *LINC01612* transcript was predominantly localized in the cytoplasmic fraction (Figure 4A). Thus, we performed RNA pull-down assay using biotin-labeled *LINC01612* and the negative control *luciferase* (*Fluc*) mRNA in DE cells to identify its interacting proteins (Figure S5A). The whole immunoprecipitated extracts of *LINC01612* and *Fluc* were then subjected to label-free quantitative mass spectrometry (Table S4). We identified 296 potential interacting proteins, among which DVL2, a WNT-associated protein, was found to directly interact with *LINC01612* (Figure 4B). The interaction of *LINC01612* with DVL2 was confirmed by western blot analysis following RNA pull-down (Figure 4C). Moreover, RNA immunoprecipitation (RIP) assay further confirmed the interaction between *LINC01612* and DVL2 (Figure 4D). These results indicate that *LINC01612* interacts with DVL2 protein.

To identify the direct binding region between *LINC01612* and DVL2, we first employed RNAfold (http://rna.tbi.univie.ac.at/cgi-bin/RNAWebSuite/RNAfold.cgi) to predict the secondary structure of *LINC01612* (Figure 4E). Based on the predicted secondary structure, we divided *LINC01612* into three fragments: 1–400 nucleotides (nts), 401–800 nts, and 801–1,045 nts. We then generated biotin-labeled truncated fragments of *LINC01612* for RNA pull-down assays in HEK293T cells. The results revealed that the 1-400-nt and 401-800-nt regions of *LINC01612* can significantly bind to DVL2 (Figure 4E). We further truncated the 1-400-nt and 401-800-nt regions of *LINC01612* into four smaller fragments and found that the fragments of 201–400 nts and 401–600 nts were primarily responsible for the interaction between *LINC01612* and DVL2 (Figure 4E). On the other hand, we truncated the DVL2 protein based on its domain annotation, which includes an N-terminal DIX domain, a central PDZ domain, and a C-terminal DEP domain (Boutros and Mlodzik, 1999). Each truncated construct was FLAG tagged and co-expressed with *LINC01612* in HEK293T cells (Figures 4F and S5B). Subsequent RIP-qPCR results showed that *LINC01612* was most

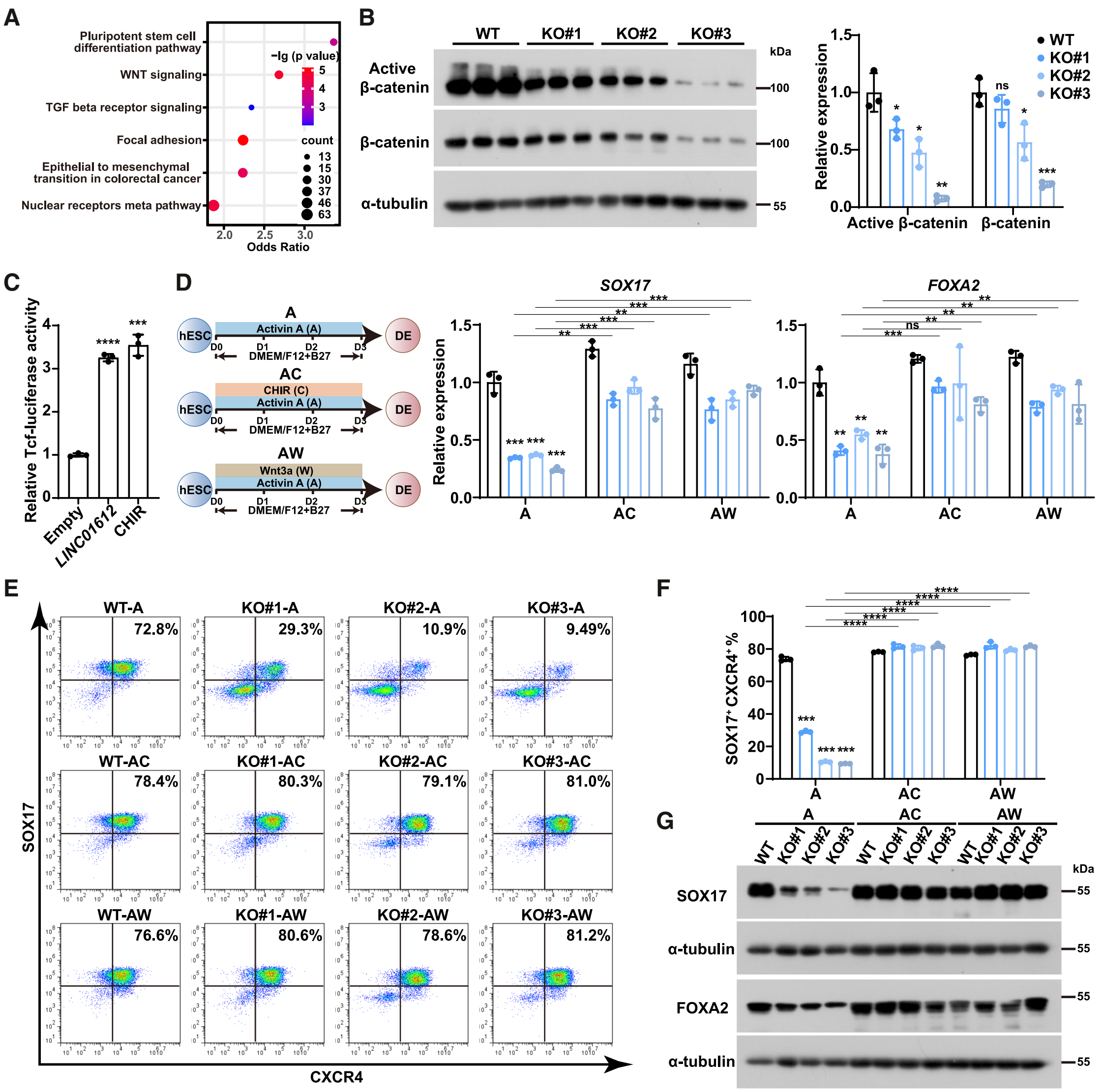

Figure 3. WNT signaling pathway acts as the downstream of *LINC01612*

(A) GO enrichment analysis of differentially expressed genes in wild-type and *LINC01612*-KO DE cells.

(B) The levels of active β-catenin and total β-catenin in wild-type and *LINC01612*-KO DE cells, determined by western blot. Quantitative results were shown on the right ($n = 3$ independent experiments).

(C) The TCF-luciferase activity in HEK293T cells transfected with *LINC01612*. The sample treated with 1 μM CHIR-99021 was used as a positive control ($n = 3$ independent experiments).

(D) Left panel: schematic representation of endoderm differentiation of human ESCs. A, Activin A; AC, Activin A and CHIR-99021; AW, Activin A and Wnt3a. Right panel: the mRNA levels of endoderm marker genes, including *SOX17* and *FOXA2*, detected by RT-qPCR ($n = 3$ independent experiments).

(E and F) Flow cytometric analysis of SOX17-and CXCR4-positive cells (E) in wild-type and *LINC01612*-KO DE cells treated with WNT activators during DE differentiation. The statistical results of double-positive cells (F) were shown ($n = 3$ independent experiments).

(G) The protein levels of DE markers (SOX17 and FOXA2) in wild-type and *LINC01612*-KO DE cells treated with WNT activators during DE differentiation, determined by western blot.

Data are presented as the mean ± SD. Significance levels are indicated as *$p < 0.05$, **$p < 0.01$, ***$p < 0.001$ and ****$p < 0.0001$; ns, not significant.

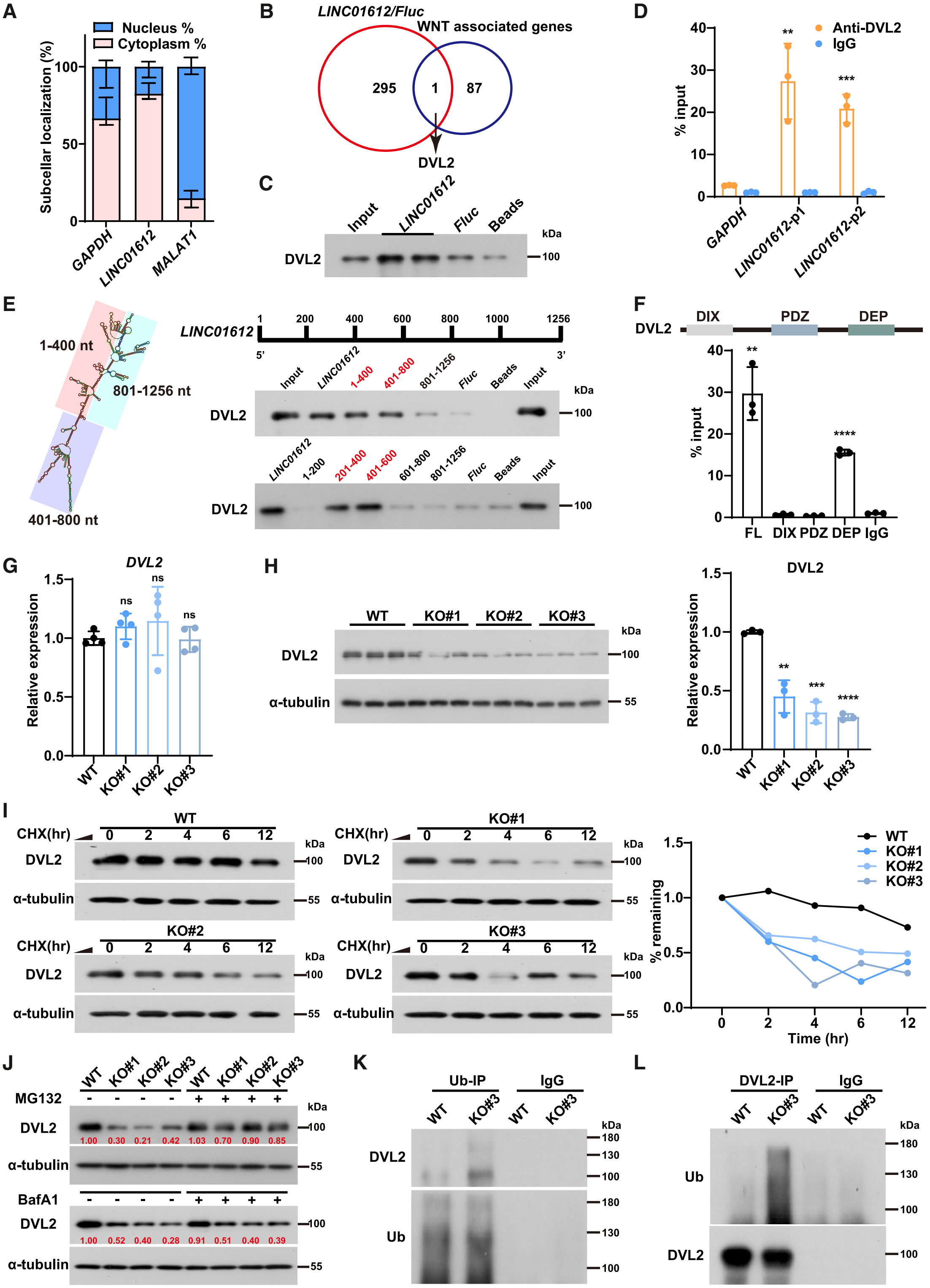

Figure 4. *LINC01612* physically interacts with and stabilizes DVL2 protein by reducing its ubiquitination

(A) Subcellular localization of *LINC01612* in DE cells by RT-qPCR (n = 6 independent experiments).

(B) Venn diagram indicating the overlapped hits identified by label-free quantitative mass spectrometry ($\log_2$(fold-change of *LINC01612*/*Fluc*) > 1) and WNT-associated genes.

(legend continued on next page)

highly enriched in the DVL2 truncation containing the DEP domain, compared to the other mutants (Figure 4F). These results suggest that the 201- to 600-nt fragment of *LINC01612* and the DEP domain of DVL2 contribute to the physical interaction with each other.

DVL2, as a component of the WNT signaling pathway, plays a pivotal role in stabilizing β-catenin by disrupting the β-catenin degradation complex (Kang et al., 2022). The time course analysis showed that DVL2 exhibited a progressive upregulation during DE differentiation (Figure S5C). To test whether *LINC01612* regulates DVL2, we examined the mRNA and protein levels of DVL2 in *LINC01612*-KO DE cells. Interestingly, although RT-qPCR results showed that the mRNA level of *DVL2* was unaltered (Figures 4G and S5D), the protein level of DVL2 was significantly downregulated in *LINC01612*-KO DE cells compared to wild-type (Figure 4H). Meanwhile, we observed a significant increase in DVL2 protein levels, but not in its mRNA expression, upon *LINC01612* overexpression in HEK293T cells (Figures S5E and S5F). These results indicate that *LINC01612* may interact with DVL2 protein and regulate its protein stability.

To confirm the protein stability regulation of DVL2 by *LINC01612*, we performed the protein synthesis inhibitor cycloheximide chase assays, revealing that *LINC01612*-KO indeed reduced DVL2 stability in DE cells (Figure 4I). This conclusion was further confirmed by the result that *LINC01612* overexpression enhanced DVL2 stability in HEK293T cells (Figure S5G). Since protein degradation is primarily mediated by the ubiquitin-proteasome system or the autophagy-lysosome pathway (Pohl and Dikic, 2019), we subsequently employed proteasome inhibitor MG132 or lysosome inhibitor bafilomycin A1 (BafA1) to elucidate the mechanism underlying DVL2 degradation. The results showed that MG132 obviously restored the change of endogenous DVL2 protein levels in both DE cells due to depletion of *LINC01612* and in HEK293T cells with *LINC01612* overexpression, but the lysosome inhibitor BafA1 had no effect on the expression of DVL2 (Figures 4J and S5H). In addition, both ubiquitination-immunoprecipitation (IP) and DVL2-IP assays showed that *LINC01612*-KO resulted in increased ubiquitination of DVL2 (Figures 4K and 4L). These findings indicate that *LINC01612* interacts with DVL2 protein and contributes to the stabilization of DVL2 by impeding ubiquitin-mediated proteasomal degradation.

LINC01612 regulates endoderm differentiation through DVL2

Since the involvement of the *LINC01612*-interacting protein DVL2 in DE differentiation was yet undocumented, we also explore whether DVL2 contributes to this process. We established three stable ESC lines of DVL2-KD without affecting the expression of pluripotency-associated genes (Figures S6A–S6C). Next, we subjected the DVL2-KD ESCs to DE differentiation. Intracellular flow cytometric analysis showed a significant decrease in the number of SOX17 and CXCR4 double-positive cells (Figure 5A). Consistently, RT-qPCR analysis showed a corresponding decrease in the expression of endodermal genes (*SOX17*, *FOXA2*, and *CXCR4*), along with elevated levels of pluripotent genes (*OCT4*, *SOX2*, and *NANOG*) and disrupted expression of mesodermal genes (*EOMES* and *T*) in DVL2-KD DE cells (Figure S6D). Furthermore, differentiated DE cells upon DVL2 depletion exhibited decreased SOX17, FOXA2, and active and total β-catenin protein levels (Figures 5B and 5C), which phenocopied the *LINC01612* depletion. These results together confirm the essential role of DVL2 in endoderm fate determination.

Next, we performed RNA-seq using DVL2-KD and control DE cells and identified 681 downregulated genes and 1,442 upregulated genes (Table S2). Consistent with RT-qPCR results (Figure S6D), the DE marker genes (*SOX17*, *FOXA2*, and *CXCR4*) were downregulated, while the

(C) Immunoblot for DVL2 after *LINC01612* RNA pull-down in DE cells. Beads and *Fluc* were used as negative controls.
(D) RIP assays showed that DVL2 enriched *LINC01612* in DE cells ($n = 3$ independent experiments).
(E) Left: the secondary structure of *LINC01612*, predicted via RNAfold. Right: immunoblot for DVL2 after truncated *LINC01612* fragment RNA pull-down in HEK293T cells.
(F) RIP assays were performed to identify the regions in DVL2 that mediate their interactions with *LINC01612* in HEK293T cells transfected with FLAG-tagged DVL2 and truncation mutants of DVL2.
(G) The RNA levels of *DVL2* in wild-type and *LINC01612*-KO DE cells, determined by RT-qPCR ($n = 4$ independent experiments).
(H) The protein levels of DVL2 in wild-type and *LINC01612*-KO DE cells, determined by western blot analysis. Quantitative results were shown on the right ($n = 3$ independent experiments).
(I) Effect of *LINC01612* on the endogenous protein levels of DVL2 in wild-type and *LINC01612*-KO DE cells treated with cycloheximide. Quantitative results were shown on the right.
(J) Effect of *LINC01612* on the endogenous protein levels of DVL2 in wild-type and *LINC01612*-KO DE cells treated with MG132 or BafA1.
(K and L) Ubiquitination-immunoprecipitation (K) and DVL2-IP (L) assays, showing the ubiquitination levels of DVL2 in wild-type and *LINC01612*-KO DE cells.
Data are presented as the mean ± SD. Significance levels are indicated as $^{**}p < 0.01$, $^{***}p < 0.001$ and $^{****}p < 0.0001$; ns, not significant.

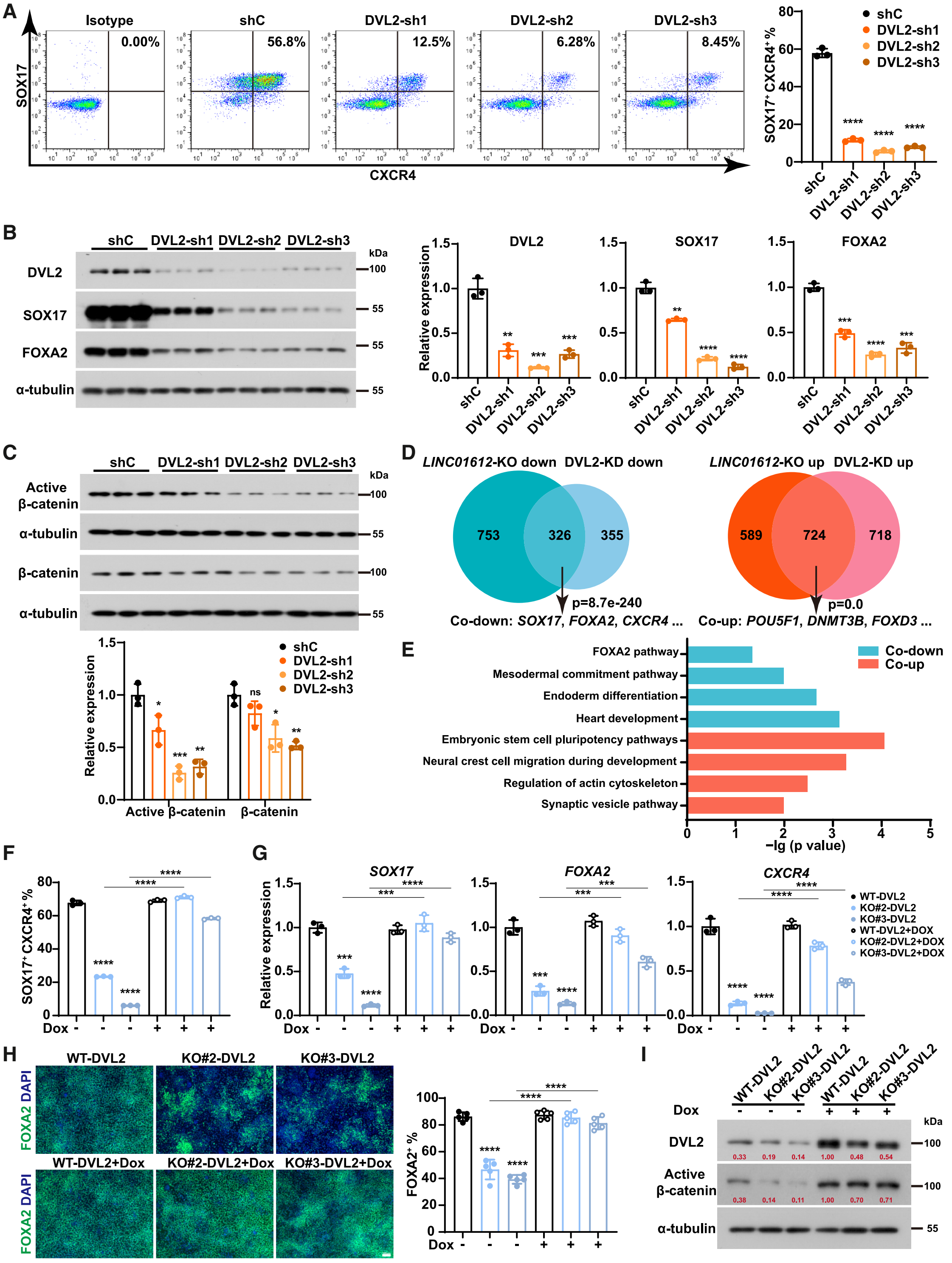

Figure 5. DVL2 counteracts the effects of *LINC01612* deficiency

(A) Flow cytometric analysis of SOX17- and CXCR4-positive cells in shRNA control (shC) and DVL2-KD DE cells. The statistical results were shown on the right (n = 3 independent experiments).

(B) The protein levels of DVL2, SOX17, and FOXA2 in DVL2-KD and control DE cells. Quantitative results were shown on the right (n = 3 independent experiments).

(legend continued on next page)

pluripotent genes (*POU5F1* and *NANOG*) were upregulated upon DVL2-KD (Figure S6E). GO analysis further showed downregulated genes in DVL2-KD DE cells enriched in terms including endoderm differentiation, WNT signaling, heart development, and EMT (Figure S6F and Table S3), similar to the results in *LINC01612*-KO DE cells (Figure 2H). GSEA also indicated the downregulation of DE-specific genes and upregulation of ESC-specific genes upon DVL2 KD (Figure S6G). To further investigate the relationship between *LINC01612* and DVL2, we performed an integrated analysis of RNA-seq datasets derived from *LINC01612*-KO and DVL2-KD DE cells. We found that there was a significant overlap between *LINC01612*- and DVL2-regulated genes, with 326 genes co-downregulated, including DE marker genes (*SOX17*, *FOXA2*, and *CXCR4*), and 724 genes co-upregulated, encompassing pluripotent genes (*POU5F1*, *DNMT3B*, and *FOXD3*) (Figure 5D). Furthermore, GO analysis revealed that co-downregulated genes enriched in terms of FOXA2 pathway, mesodermal commitment pathway, and endoderm differentiation. In contrast, the co-upregulated genes were associated with ESC pluripotency pathways, neural crest cell migration during development, regulation of actin cytoskeleton, and synaptic vesicle pathway (Figure 5E and Table S3). In addition to their cooperative role in endoderm differentiation, *LINC01612* and DVL2 also independently regulate distinct sets of genes involved in various metabolic and signaling pathways (Figure S6H). These findings suggest that *LINC01612* and DVL2 function within the same pathway to influence endoderm differentiation.

To determine whether *LINC01612* exerts its function through DVL2, we performed DVL2 overexpression experiments in *LINC01612*-KO cells. Significantly, DVL2 overexpression effectively restored DE differentiation in *LINC01612*-KO cells and rescued the expression of DE markers at both mRNA and protein levels (Figures 5F–5H and S6I). Additionally, DVL2 overexpression was able to increase the active β-catenin protein level and rescue WNT signaling activity (Figure 5I). Altogether, these data demonstrate that *LINC01612* enhances WNT signaling activity and DE differentiation via stabilizing DVL2 protein (Figure 6).

DISCUSSION

This study dissects the biological function and molecular mechanisms of a new desert lncRNA *LINC01612* during human endoderm differentiation. Our findings demonstrate that as a highly expressed lncRNA during DE differentiation, *LINC01612* is essential for the process, evident by the data that the disruption through shRNA KD or promoter deletion significantly impairs DE differentiation (Figures 1 and 2). Furthermore, depletion of *LINC01612* leads to reduced WNT signaling activity, coupled with the restoration of impaired DE differentiation by WNT signaling activators, strongly suggesting that the WNT signaling pathway serves as the functional downstream of *LINC01612* in promoting DE differentiation (Figure 3). Mechanistically, we show that *LINC01612* physically interacts with and stabilizes DVL2 protein by reducing its ubiquitination (Figure 4). DVL2-KD results in suppression of endoderm differentiation. At the same time, DVL2 overexpression rescues the defects caused by *LINC01612* deficiency, further demonstrating that DVL2 is the functional target of *LINC01612* (Figure 5). Our study highlights the critical regulatory role of *LINC01612*-DVL2-WNT axis in human endoderm differentiation (Figure 6).

LncRNAs are transcribed by RNA polymerase II and lack long and conserved ORFs (Chen and Kim, 2024). However, it has been increasingly recognized that a subset of lncRNAs containing smORFs can give rise to micropeptides (Orr et al., 2020). In our results, *LINC01612* contains four predicted smORFs, and the exogenous overexpression of ORF-EGFP fusion proteins revealed that ORF1 and ORF3 showed the capacity to be translated into micropeptides (Figures S1C–S1F). However, further *in situ* EGFP knockin experiments demonstrated that neither produced micropeptides endogenously (Figures 1F, 1G, and S1G). This suggests that utilizing a strong promoter for ORF-EGFP overexpression markedly increases its expression level and that the differences in cellular environments do not accurately reflect the endogenous translation events. Therefore, a comprehensive approach is crucial for determining whether lncRNAs can be translated into micropeptides,

(C) The levels of active β-catenin and total β-catenin in DVL2-KD and control DE cells, determined by western blot. Quantitative results were shown at the bottom (n = 3 independent experiments).
(D) Venn diagram indicating the significant overlap of differentially expressed genes due to *LINC01612*-KO and DVL2-KD in DE cells.
(E) GO enrichment analysis of overlapped differentially expressed genes in *LINC01612*-KO and DVL2-KD DE cells.
(F) Flow cytometric analysis of the SOX17-/CXCR4-double positive cells in differentiated wild-type, *LINC01612*-KO, and DVL2-overexpressing cells with *LINC01612*-KO (n = 3 independent experiments).
(G) The RNA levels of endoderm genes (*SOX17*, *FOXA2*, and *CXCR4*) for the samples shown in (F) (n = 3 independent experiments).
(H) Immunofluorescent staining of FOXA2 for the samples shown in (F). Quantitative results were shown on the right (n = 5 images). Scale bar, 100 μm.
(I) Western blot analysis of DVL2 and active β-catenin levels for the samples shown in (F).
Data are presented as the mean ± SD. Significance levels are indicated as $^{**}p < 0.01$, $^{***}p < 0.001$ and $^{****}p < 0.0001$; ns, not significant.

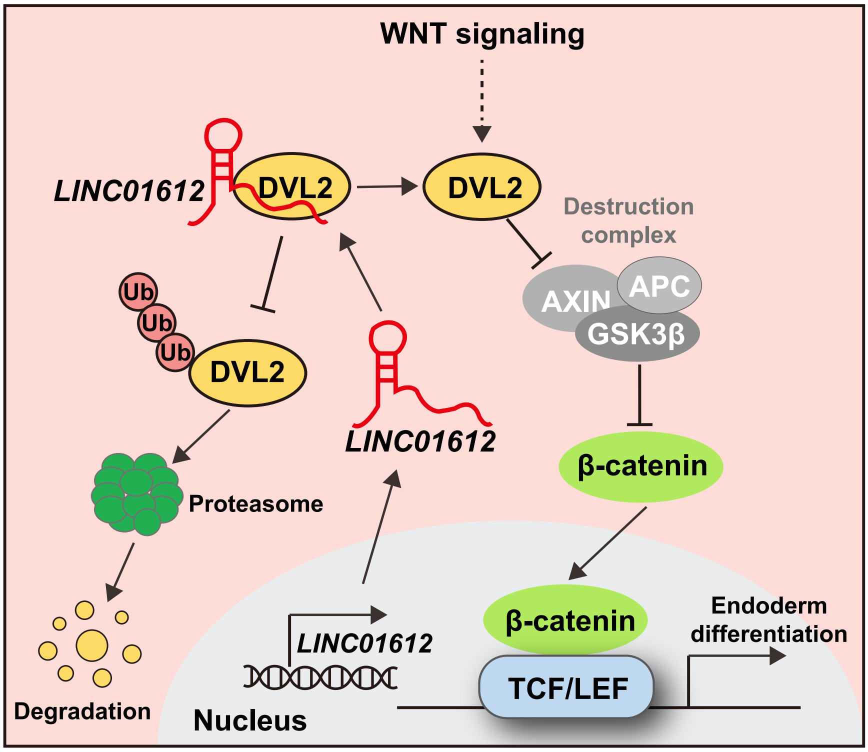

Figure 6. Model of the role of *LINC01612*-DVL2-WNT axis in endoderm differentiation

During human endoderm differentiation, *LINC01612* enhances WNT signaling activity by binding to DVL2 protein, reducing its ubiquitination, and thereby protecting DVL2 protein from proteasomal degradation.

combining bioinformatic analysis, ribosome profiling, mass spectrometry, proteomics, monoclonal antibodies, and *in situ* fusion protein detection.

We have noticed that *LINC01612* might have other functions in different biological contexts. An expression analysis suggested that *LINC01612* is significantly downregulated in gastric cancer samples and closely linked to enhanced metastasis and advanced tumor stage (Song et al., 2016). Moreover, *LINC01612* inhibits gastric cancer cell proliferation, induces apoptosis by binding to ATR and suppressing CHK1 phosphorylation, and enhances oxaliplatin sensitivity (Liu et al., 2024). In p53-expressing hepatocellular carcinoma cells, *LINC01612* promotes ATF3 expression by sponging miR-494 and activating the p53 pathway, while in p53-null cells, *LINC01612* binds to YBX1 and promotes its degradation to suppress tumor progression (Liu et al., 2022). In our case, *LINC01612* stabilizes DVL2 protein, a key WNT pathway regulator, by reducing its ubiquitination, thereby playing a crucial role in the differentiation process. Such cell-type specificity stems from the distinct expression patterns of lncRNAs, their interactions with different proteins, and their involvement in signaling pathways adapted to cellular contexts. Whether the *LINC01612*-DVL2-WNT regulatory axis is conserved in certain developmental or pathological contexts involving WNT signaling awaits further investigation.

DVL2 is an essential modulator of the WNT signaling pathway, playing a role in maintaining β-catenin stability by disrupting the degradation complex formed by APC, AXIN, CK1α, and GSK3β (Kang et al., 2022; Schubert et al., 2022). In addition, DVL2/WNT/β-catenin axis has been identified as a driver of progression mediated by KLF12 in pancreatic cancer (He et al., 2019). Here, we discovered that depleting DVL2 impairs WNT signaling activity, subsequently affecting endoderm differentiation (Figure 5). Of note, in addition to their cooperative role in endoderm differentiation, *LINC01612* and DVL2 independently regulate distinct sets of genes involved in various metabolic and signaling pathways as well (Figure S6H). Specifically, Qu and colleagues demonstrated the downregulation of genes in the Hippo/YAP signaling pathway and upregulation of genes involved in cell metabolism upon macrophage DVL2 deficiency (Qu et al., 2025), aligning well with our results. This consistency reinforces the role of DVL2 in regulating these pathways and supports the biological relevance of our observations.

Increasing evidence suggests that multiple E3 ligases, such as NEDD4L, ITCH, and TRIM56, bind to the C terminus of DVL2 and mediate DVL2 ubiquitination, ultimately promoting its degradation and attenuating WNT signaling activity (Ding et al., 2013; Wei et al., 2012; Yan et al., 2021). Meanwhile, USP14 acts as a deubiquitinating enzyme for DVL, facilitating its deubiquitylation and consequently enhancing WNT signaling activity (Jung et al., 2013). Our results showed that *LINC01612* interacts with DVL2 via the 201- to 600-nt fragment of *LINC01612* and the DEP domain

of DVL2 (Figure 4). The DEP domain is essential for WNT signal transduction to the nucleus (Gammons et al., 2016) and harbors its ubiquitination site (Ding et al., 2013; Wei et al., 2012; Yan et al., 2021). It has been reported that lncRNAs can influence the ubiquitination of their associated proteins and regulate their stability (Bian et al., 2024). Nevertheless, it remains to be determined whether *LINC01612* affects the ubiquitination of DVL2 through their interaction by either occupying the binding site on DVL2 for E3 ubiquitin ligases, thereby preventing ubiquitination, or by recruiting deubiquitinating enzymes to DVL2 via the non-interacting region of *LINC01612* to reduce its ubiquitination. Further studies are needed to understand how *LINC01612* regulates DVL2 protein stability.

In summary, here we report the role of a newly discovered desert lncRNA, *LINC01612*, in the human endoderm differentiation. *LINC01612* binds to and stabilizes DVL2 protein, and the depletion of *LINC01612* or DVL2 compromises WNT signaling activity and abolishes endoderm differentiation. These results reveal the role and action of the desert lncRNA, *LINC01612,* in early embryonic lineage specification and provide deeper insights into the regulatory mechanisms underlying cell fate decisions.

MATERIALS AND METHODS

Cell culture and differentiation

The human ESC line HUES8 from Harvard University was maintained in mTeSR1 medium (STEMCELL Technologies, Cat#85850) and 1% PS (penicillin-streptomycin) (Gibco, Cat#10378016) on Matrigel-coated plates at 37°C with 5% CO_2. Cells were passaged every 3–4 days at 1:20 ratio by incubating cells with Accutase (Sigma, Cat#A6964) for 3 min. All the HUES8 lines tested negative for mycoplasma contamination. Our work on human ESCs is approved by the Biomedical Ethics Committee of Wuhan University (WHU-LFMD-IRB2024026). HEK293T cells were cultured in DMEM (Gibco, Cat#C11995500BT) supplemented with 10% FBS (fetal bovine serum) (Gibco, Cat#10100147) and 1% PS at 37°C with 5% CO_2.

DE differentiation was performed following a previously described protocol (Lu et al., 2023; Yang et al., 2020). Briefly, $5 \sim 8 \times 10^4$ cells were seeded onto Matrigel-coated 24-well plates in mTeSR1. On the following day, the culture medium was replaced with DE induction medium consisting of DMEM/F12 (Gibco, Cat#C11330500BT), 0.2% BSA (bovine serum albumin) (YEASEN, Cat#B57370), 1% B27 without vitamin A (Shanghai BasalMedia, Cat#S441J7), and 100 ng/mL Activin A (PeproTech, Cat#120-14P). Cells were collected on day 3 for immunofluorescence, RT-qPCR, western blot, or flow cytometric analysis. The pancreatic lineage and heart progenitor differentiation were performed according to the published differentiation protocol (Li et al., 2024; Yang et al., 2024) and EB formation was carried out as previously described (Lan et al., 2022).

Plasmid constructs of shRNA knockdown and gene overexpression

The shRNAs targeting *LINC01612*, DVL2, and a scramble control were inserted into the lentiviral vector pLKO.1 plasmid. HEK293T cells were co-transfected with these lentiviral plasmids, expressing either the specific shRNAs or the scramble control (shC), along with lentiviral packaging plasmids (psPAX2 and pMD2.G) for lentivirus production. DVL2 CDS sequence was inserted into the doxycycline-induced pCW plasmid and co-transfected with lentiviral packaging plasmids into *LINC01612*-KO ESCs cells. Stable ESC lines were established through selection with 2 μg/mL puromycin for about 7 days. The targeting sequences were provided in Table S5.

CRISPR-Cas9-mediated knockout and knockin in human ESCs

Pairwise guide RNAs (gRNAs) targeting *LINC01612* were designed using CHOPCHOP web tool (https://chopchop.cbu.uib.no/), and they were constructed in pX459 (pSpCas9 (BB)-2A-Puro, Addgene, Cat#48139) expression plasmid. As for knockin, about 2,000-bp double-stranded donor DNA was gel-purified from the PCR products of genome sequence carrying EGFP. Briefly, 1 million HUES8 cells were electroporated with a total of 10 μg pX459 and donor DNA. The cells were then seeded onto a Matrigel-coated 6-well plate in mTesR1 medium supplemented with 10 μM Y-27632 (Selleck, Cat#S1049). After 24 h of culture, puromycin (1 μg/mL) (Santa Cruz Biotechnology, Cat#58582) was added for an additional 48 h. Single cells were subsequently sorted into Matrigel-coated 96-well plates using FACSAria III. Surviving colonies were expanded and genotyped through Sanger sequencing. The sequences of all sgRNAs and the primers for genomic sequencing were listed in Table S5.

RNA-seq and data analysis

Total RNA was sent to YINGZI GENE (Wuhan, China) for library preparation and sequencing. RNA-seq data were aligned to the human genome (hg38) using HISAT2 (Kim et al., 2019). FeatureCounts (v2.0.1) was used to count reads based on the GENCODE V29 gene transfer format, and gene expression was quantified using transcripts per million (TPM). Differential gene expression analysis for binary comparisons was carried out using the DESeq2 R package, with a fold-change cutoff >1, p value <0.05, and average TPM>10 (Love et al., 2014). GO analysis was performed using Enrichr (https://maayanlab.cloud/Enrichr/) (Kuleshov et al., 2016).

Statistical analysis

Data were presented as means ± SD from at least three independent experiments. The significance levels were determined using an unpaired t test in GraphPad Prism 9.5. p values were shown as *$p < 0.05$, **$p < 0.01$, ***$p < 0.001$, and ****$p < 0.0001$, with "ns" indicating no statistical significance.

RESOURCE AVAILABILITY

Lead contact

Further information and requests for resources and reagents should be directed to the lead contact, Wei Jiang (jiangw.mri@whu.edu.cn).

Materials availability

Plasmids and cell lines generated in this study are available from the lead contact upon request.

Data and code availability

The RNA-seq data in this study have been uploaded to the Gene Expression Omnibus (GEO) database under accession code GEO: GSE291901.

ACKNOWLEDGMENTS

We would like to thank laboratory members for technical help and insightful discussion. We thank the Core Facility of Medical Research Institute of Wuhan University and Large-Scale Instrument and Equipment Sharing Foundation of Wuhan University for technical support and equipment grant. This work was supported by grants from the National Natural Science Foundation of China (No. 32270857 to W.J. and No. 32400672 to P.L.), the China Postdoctoral Science Foundation (GZC20251853 and 2025M772807 to J.Y.) and the Fundamental Research Funds for the Central Universities in China (2042022dx0003 to W.J.).

AUTHOR CONTRIBUTIONS

W.J. conceived the project and designed the experiment together with M.L., P.L., and J.Y.; M.L. performed most of the bench experiments with help from P.L. and J.Y.; P.L. performed the initial screening, *LINC01612*-KD, and nucleo/cytoplasmic separation experiments; J.Y. performed the 5′ and 3′ RACE experiments; Y.Y. performed the ORF-EGFP experiments; C.Y. and J.Y. analyzed the next-generation sequencing data; M.L. drafted the manuscript, and W.J. and M.L. finalized the manuscript. All authors contributed to and approved the final manuscript.

DECLARATION OF INTERESTS

The authors declare no competing interests.

SUPPLEMENTAL INFORMATION

Supplemental information can be found online at https://doi.org/10.1016/j.stemcr.2025.102682.

Received: May 14, 2025
Revised: September 23, 2025
Accepted: September 25, 2025
Published: October 23, 2025

REFERENCES

Amodio, N., Raimondi, L., Juli, G., Stamato, M.A., Caracciolo, D., Tagliaferri, P., and Tassone, P. (2018). MALAT1: a druggable long non-coding RNA for targeted anti-cancer approaches. J. Hematol. Oncol. *11*, 63. https://doi.org/10.1186/s13045-018-0606-4.

Andrews, S.J., and Rothnagel, J.A. (2014). Emerging evidence for functional peptides encoded by short open reading frames. Nat. Rev. Genet. *15*, 193–204. https://doi.org/10.1038/nrg3520.

Ang, S.L., Wierda, A., Wong, D., Stevens, K.A., Cascio, S., Rossant, J., and Zaret, K.S. (1993). The formation and maintenance of the definitive endoderm lineage in the mouse: involvement of HNF3/forkhead proteins. Development *119*, 1301–1315. https://doi.org/10.1242/dev.119.4.1301.

Bian, Z., Yang, F., Xu, P., Gao, G., Yang, C., Cao, Y., Yao, S., Wang, X., Yin, Y., Fei, B., and Huang, Z. (2024). LINC01852 inhibits the tumorigenesis and chemoresistance in colorectal cancer by suppressing SRSF5-mediated alternative splicing of PKM. Mol. Cancer *23*, 23. https://doi.org/10.1186/s12943-024-01939-7.

Boutros, M., and Mlodzik, M. (1999). Dishevelled: at the crossroads of divergent intracellular signaling pathways. Mech. Dev. *83*, 27–37. https://doi.org/10.1016/s0925-4773(99)00046-5.

Chen, L.L., and Kim, V.N. (2024). Small and long non-coding RNAs: Past, present, and future. Cell *187*, 6451–6485. https://doi.org/10.1016/j.cell.2024.10.024.

D'Amour, K.A., Agulnick, A.D., Eliazer, S., Kelly, O.G., Kroon, E., and Baetge, E.E. (2005). Efficient differentiation of human embryonic stem cells to definitive endoderm. Nat. Biotechnol. *23*, 1534–1541. https://doi.org/10.1038/nbt1163.

D'Lima, N.G., Ma, J., Winkler, L., Chu, Q., Loh, K.H., Corpuz, E.O., Budnik, B.A., Lykke-Andersen, J., Saghatelian, A., and Slavoff, S.A. (2017). A human microprotein that interacts with the mRNA decapping complex. Nat. Chem. Biol. *13*, 174–180. https://doi.org/10.1038/nchembio.2249.

Daneshvar, K., Ardehali, M.B., Klein, I.A., Hsieh, F.K., Kratkiewicz, A.J., Mahpour, A., Cancelliere, S.O.L., Zhou, C., Cook, B.M., Li, W., et al. (2020). lncRNA DIGIT and BRD3 protein form phase-separated condensates to regulate endoderm differentiation. Nat. Cell Biol. *22*, 1211–1222. https://doi.org/10.1038/s41556-020-0572-2.

Daneshvar, K., Pondick, J.V., Kim, B.M., Zhou, C., York, S.R., Macklin, J.A., Abualteen, A., Tan, B., Sigova, A.A., Marcho, C., et al. (2016). DIGIT Is a Conserved Long Noncoding RNA that Regulates GSC Expression to Control Definitive Endoderm Differentiation of Embryonic Stem Cells. Cell Rep. *17*, 353–365. https://doi.org/10.1016/j.celrep.2016.09.017.

Ding, Y., Zhang, Y., Xu, C., Tao, Q.H., and Chen, Y.G. (2013). HECT domain-containing E3 ubiquitin ligase NEDD4L negatively regulates Wnt signaling by targeting dishevelled for proteasomal degradation. J. Biol. Chem. *288*, 8289–8298. https://doi.org/10.1074/jbc.M112.433185.

Dziedzicka, D., Tewary, M., Keller, A., Tilleman, L., Prochazka, L., Östblom, J., Couvreu De Deckersberg, E., Markouli, C., Franck, S., Van Nieuwerburgh, F., et al. (2021). Endogenous suppression of WNT signalling in human embryonic stem cells leads to low differentiation propensity towards definitive endoderm. Sci. Rep. *11*, 6137. https://doi.org/10.1038/s41598-021-85447-4.

Flynn, R.A., and Chang, H.Y. (2014). Long noncoding RNAs in cell-fate programming and reprogramming. Cell Stem Cell *14*, 752–761. https://doi.org/10.1016/j.stem.2014.05.014.

Gammons, M.V., Renko, M., Johnson, C.M., Rutherford, T.J., and Bienz, M. (2016). Wnt Signalosome Assembly by DEP Domain Swapping of Dishevelled. Mol. Cell *64*, 92–104. https://doi.org/10.1016/j.molcel.2016.08.026.

Gil, N., and Ulitsky, I. (2020). Regulation of gene expression by cis-acting long non-coding RNAs. Nat. Rev. Genet. *21*, 102–117. https://doi.org/10.1038/s41576-019-0184-5.

He, Z., Guo, X., Tian, S., Zhu, C., Chen, S., Yu, C., Jiang, J., and Sun, C. (2019). MicroRNA-137 reduces stemness features of pancreatic cancer cells by targeting KLF12. J. Exp. Clin. Cancer Res. *38*, 126. https://doi.org/10.1186/s13046-019-1105-3.

Heslop, J.A., Pournasr, B., Liu, J.T., and Duncan, S.A. (2021). GATA6 defines endoderm fate by controlling chromatin accessibility during differentiation of human-induced pluripotent stem cells. Cell Rep. *35*, 109145. https://doi.org/10.1016/j.celrep.2021.109145.

Jiang, W., Liu, Y., Liu, R., Zhang, K., and Zhang, Y. (2015). The lncRNA DEANR1 facilitates human endoderm differentiation by activating FOXA2 expression. Cell Rep. *11*, 137–148. https://doi.org/10.1016/j.celrep.2015.03.008.

Jiang, W., Wang, J., and Zhang, Y. (2013). Histone H3K27me3 demethylases KDM6A and KDM6B modulate definitive endoderm differentiation from human ESCs by regulating WNT signaling pathway. Cell Res. *23*, 122–130. https://doi.org/10.1038/cr.2012.119.

Jung, H., Kim, B.G., Han, W.H., Lee, J.H., Cho, J.Y., Park, W.S., Maurice, M.M., Han, J.K., Lee, M.J., Finley, D., and Jho, E.H. (2013). Deubiquitination of Dishevelled by Usp14 is required for Wnt signaling. Oncogenesis *2*, e64. https://doi.org/10.1038/oncsis.2013.28.

Kang, K., Shi, Q., Wang, X., and Chen, Y.G. (2022). Dishevelled phase separation promotes Wnt signalosome assembly and destruction complex disassembly. J. Cell Biol. *221*, e202205069. https://doi.org/10.1083/jcb.202205069.

Kim, D., Paggi, J.M., Park, C., Bennett, C., and Salzberg, S.L. (2019). Graph-based genome alignment and genotyping with HISAT2 and HISAT-genotype. Nat. Biotechnol. *37*, 907–915. https://doi.org/10.1038/s41587-019-0201-4.

Kopp, F., and Mendell, J.T. (2018). Functional Classification and Experimental Dissection of Long Noncoding RNAs. Cell *172*, 393–407. https://doi.org/10.1016/j.cell.2018.01.011.

Kuleshov, M.V., Jones, M.R., Rouillard, A.D., Fernandez, N.F., Duan, Q., Wang, Z., Koplev, S., Jenkins, S.L., Jagodnik, K.M., Lachmann, A., et al. (2016). Enrichr: a comprehensive gene set enrichment analysis web server 2016 update. Nucleic Acids Res. *44*, W90–W97. https://doi.org/10.1093/nar/gkw377.

Lan, X., Ding, S., Zhang, T., Yi, Y., Li, C., Jin, W., Chen, J., Liang, K., Wang, H., and Jiang, W. (2022). PCGF6 controls neuroectoderm specification of human pluripotent stem cells by activating SOX2 expression. Nat. Commun. *13*, 4601. https://doi.org/10.1038/s41467-022-32295-z.

Li, Y., Zheng, R., Jiang, L., Yan, C., Liu, R., Chen, L., Jin, W., Luo, Y., Zhang, X., Tang, J., et al. (2024). A noncoding variant confers pancreatic differentiation defect and contributes to diabetes susceptibility by recruiting RXRA. Nat. Commun. *15*, 9771. https://doi.org/10.1038/s41467-024-54151-y.

Liu, P., Zhong, Q., Song, Y., Guo, D., Ma, D., Chen, B., Lan, J., and Liu, Q. (2022). Long noncoding RNA Linc01612 represses hepatocellular carcinoma progression by regulating miR-494/ATF3/p53 axis and promoting ubiquitination of YBX1. Int. J. Biol. Sci. *18*, 2932–2948. https://doi.org/10.7150/ijbs.69514.

Liu, W., Feng, W., Zhang, Y., Lei, T., Wang, X., Qiao, T., Chen, Z., and Song, W. (2024). RP11-789C1.1 inhibits gastric cancer cell proliferation and accelerates apoptosis via the ATR/CHK1 signaling pathway. Chin. Med. J. *137*, 1835–1843. https://doi.org/10.1097/CM9.0000000000002869.

Loh, K.M., Ang, L.T., Zhang, J., Kumar, V., Ang, J., Auyeong, J.Q., Lee, K.L., Choo, S.H., Lim, C.Y.Y., Nichane, M., et al. (2014). Efficient endoderm induction from human pluripotent stem cells by logically directing signals controlling lineage bifurcations. Cell Stem Cell *14*, 237–252. https://doi.org/10.1016/j.stem.2013.12.007.

Love, M.I., Huber, W., and Anders, S. (2014). Moderated estimation of fold change and dispersion for RNA-seq data with DESeq2. Genome Biol. *15*, 550. https://doi.org/10.1186/s13059-014-0550-8.

Lu, P., Li, M., Zhang, D., and Jiang, W. (2021). Lnc-ing pluripotency maintenance and early differentiation in human pluripotent stem cells. FASEB J. *35*, e21438. https://doi.org/10.1096/fj.202002278R.

Lu, P., Yang, J., Li, M., Wen, S., Zhang, T., Yan, C., Liu, R., Xiao, Y., Wang, X., and Jiang, W. (2023). A desert lncRNA HIDEN regulates human endoderm differentiation via interacting with IMP1 and stabilizing FZD5 mRNA. Genome Biol. *24*, 92. https://doi.org/10.1186/s13059-023-02925-w.

Mattick, J.S., Amaral, P.P., Carninci, P., Carpenter, S., Chang, H.Y., Chen, L.L., Chen, R., Dean, C., Dinger, M.E., Fitzgerald, K.A., et al. (2023). Long non-coding RNAs: definitions, functions, challenges and recommendations. Nat. Rev. Mol. Cell Biol. *24*, 430–447. https://doi.org/10.1038/s41580-022-00566-8.

Mirzadeh Azad, F., Polignano, I.L., Proserpio, V., and Oliviero, S. (2021). Long Noncoding RNAs in Human Stemness and Differentiation. Trends Cell Biol. *31*, 542–555. https://doi.org/10.1016/j.tcb.2021.02.002.

Orr, M.W., Mao, Y., Storz, G., and Qian, S.B. (2020). Alternative ORFs and small ORFs: shedding light on the dark proteome. Nucleic Acids Res. *48*, 1029–1042. https://doi.org/10.1093/nar/gkz734.

Pang, Y., Mao, C., and Liu, S. (2018). Encoding activities of non-coding RNAs. Theranostics *8*, 2496–2507. https://doi.org/10.7150/thno.24677.

Pohl, C., and Dikic, I. (2019). Cellular quality control by the ubiquitin-proteasome system and autophagy. Science *366*, 818–822. https://doi.org/10.1126/science.aax3769.

Qu, X., Xu, D., Yang, T., Tian, Y., King, C.T., Wang, X., Sheng, M., Lin, Y., Bian, X., Li, C., et al. (2025). Macrophage Dvl2 deficiency promotes NOD1-Driven pyroptosis and exacerbates inflammatory liver injury. Redox Biol. *79*, 103455. https://doi.org/10.1016/j.redox.2024.103455.

Quinn, J.J., and Chang, H.Y. (2016). Unique features of long non-coding RNA biogenesis and function. Nat. Rev. Genet. *17*, 47–62. https://doi.org/10.1038/nrg.2015.10.

Schubert, A., Voloshanenko, O., Ragaller, F., Gmach, P., Kranz, D., Scheeder, C., Miersch, T., Schulz, M., Trümper, L., Binder, C., et al. (2022). Superresolution microscopy localizes endogenous Dvl2 to Wnt signaling-responsive biomolecular condensates. Proc. Natl. Acad. Sci. USA *119*, e2122476119. https://doi.org/10.1073/pnas.2122476119.

Song, W., Liu, Y.Y., Peng, J.J., Liang, H.H., Chen, H.Y., Chen, J.H., He, W.L., Xu, J.B., Cai, S.R., and He, Y.L. (2016). Identification of differentially expressed signatures of long non-coding RNAs associated with different metastatic potentials in gastric cancer. J. Gastroenterol. *51*, 119–129. https://doi.org/10.1007/s00535-015-1091-y.

St Laurent, G., Wahlestedt, C., and Kapranov, P. (2015). The Landscape of long noncoding RNA classification. Trends Genet. *31*, 239–251. https://doi.org/10.1016/j.tig.2015.03.007.

Statello, L., Guo, C.J., Chen, L.L., and Huarte, M. (2021). Gene regulation by long non-coding RNAs and its biological functions. Nat. Rev. Mol. Cell Biol. *22*, 96–118. https://doi.org/10.1038/s41580-020-00315-9.

Thomson, J.A., Itskovitz-Eldor, J., Shapiro, S.S., Waknitz, M.A., Swiergiel, J.J., Marshall, V.S., and Jones, J.M. (1998). Embryonic stem cell lines derived from human blastocysts. Science *282*, 1145–1147. https://doi.org/10.1126/science.282.5391.1145.

Viotti, M., Nowotschin, S., and Hadjantonakis, A.K. (2014). SOX17 links gut endoderm morphogenesis and germ layer segregation. Nat. Cell Biol. *16*, 1146–1156. https://doi.org/10.1038/ncb3070.

Wang, K.C., and Chang, H.Y. (2011). Molecular mechanisms of long noncoding RNAs. Mol. Cell *43*, 904–914. https://doi.org/10.1016/j.molcel.2011.08.018.

Wei, W., Li, M., Wang, J., Nie, F., and Li, L. (2012). The E3 ubiquitin ligase ITCH negatively regulates canonical Wnt signaling by targeting dishevelled protein. Mol. Cell Biol. *32*, 3903–3912. https://doi.org/10.1128/mcb.00251-12.

Yan, H., Wang, Z., Sun, Y., Hu, L., and Bu, P. (2021). Cytoplasmic NEAT1 Suppresses AML Stem Cell Self-Renewal and Leukemogenesis through Inactivation of Wnt Signaling. Adv. Sci. *8*, e2100914. https://doi.org/10.1002/advs.202100914.

Yan, P., Luo, S., Lu, J.Y., and Shen, X. (2017). Cis- and trans-acting lncRNAs in pluripotency and reprogramming. Curr. Opin. Genet. Dev. *46*, 170–178. https://doi.org/10.1016/j.gde.2017.07.009.

Yang, J., Lu, P., Li, M., Yan, C., Zhang, T., and Jiang, W. (2020). GATA6-AS1 Regulates GATA6 Expression to Modulate Human Endoderm Differentiation. Stem Cell Rep. *15*, 694–705. https://doi.org/10.1016/j.stemcr.2020.07.014.

Yang, J., Zhang, D., and Jiang, W. (2025). Long noncoding RNA as an emerging regulator of endoderm differentiation: progress and perspectives. Cell Regen. *14*, 11. https://doi.org/10.1186/s13619-025-00230-4.

Yang, P., Zhu, L., Wang, S., Gong, J., Selvaraj, J.N., Ye, L., Chen, H., Zhang, Y., Wang, G., Song, W., et al. (2024). Engineered model of heart tissue repair for exploring fibrotic processes and therapeutic interventions. Nat. Commun. *15*, 7996. https://doi.org/10.1038/s41467-024-52221-9.

Yiangou, L., Ross, A.D.B., Goh, K.J., and Vallier, L. (2018). Human Pluripotent Stem Cell-Derived Endoderm for Modeling Development and Clinical Applications. Cell Stem Cell *22*, 485–499. https://doi.org/10.1016/j.stem.2018.03.016.

Zhai, J., Xiao, Z., Wang, Y., and Wang, H. (2022). Human embryonic development: from peri-implantation to gastrulation. Trends Cell Biol. *32*, 18–29. https://doi.org/10.1016/j.tcb.2021.07.008.

Zorn, A.M., and Wells, J.M. (2009). Vertebrate endoderm development and organ formation. Annu. Rev. Cell Dev. Biol. *25*, 221–251. https://doi.org/10.1146/annurev.cellbio.042308.113344.

Stem Cell Reports, Volume *20*

Supplemental Information

The *LINC01612*-DVL2-WNT axis promotes human endoderm differentiation

Mao Li, Pei Lu, Jie Yang, Chenchao Yan, Yikang Yang, and Wei Jiang

Supplemental Information

Supplementary figures and legends

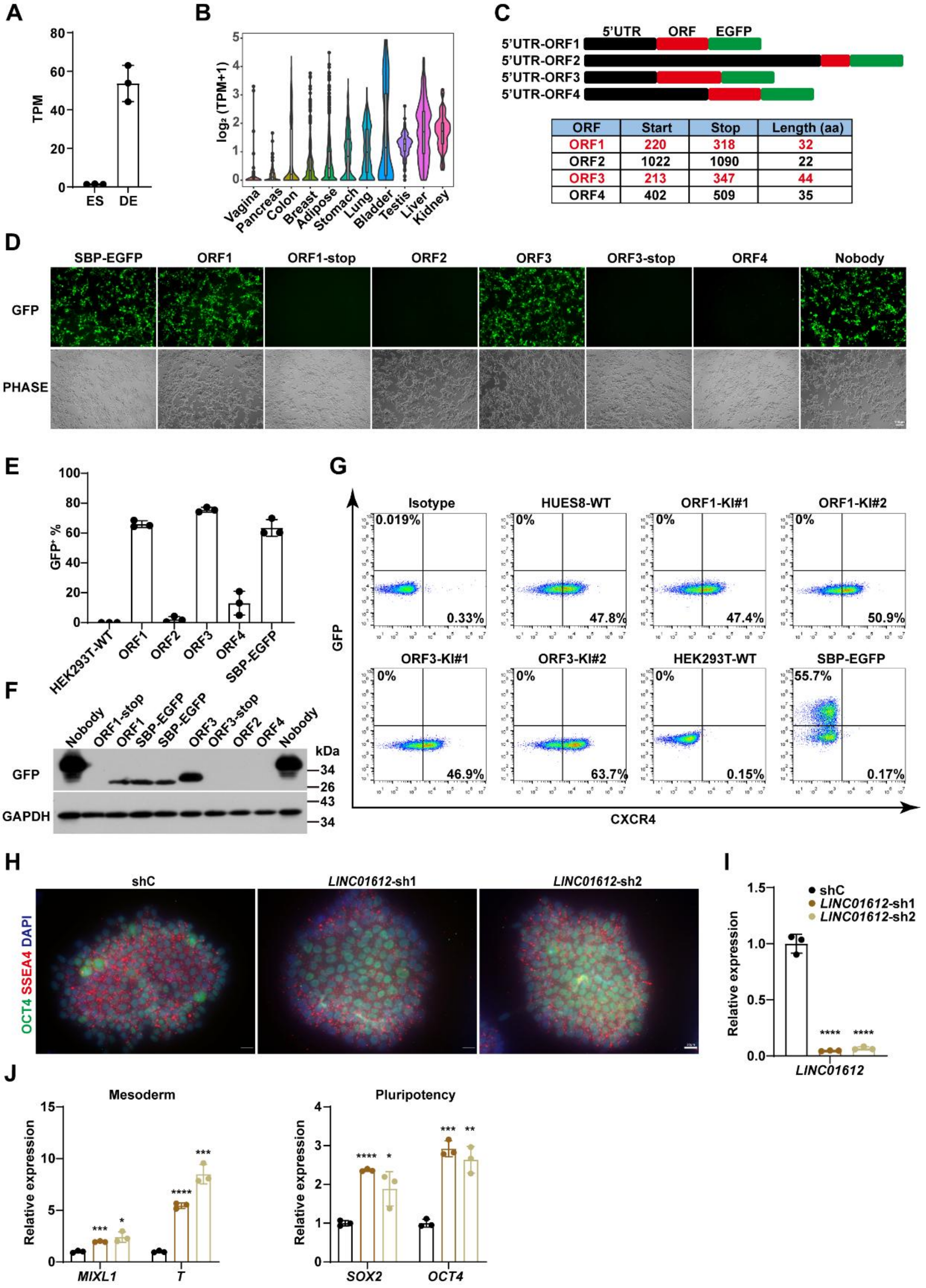

Figure S1. *LINC01612* lacks protein-coding ability, related to Fig 1.

(A) The TPM value of *LINC01612* in ESC and DE cells (date from GSE162649).

(B) The expression of *LINC01612* in 30 human tissues from GTEx database. The top eleven tissues with high expression were shown. *LINC01612* is merely expressed in the other 29 tissues (Blood, Brain,

Prostate, etc.).

(C) Predicted ORFs of *LINC01612* and diagram of the ORFs constructs with C-terminal EGFP-tag.

(D-E) Expression of ORFs-EGFP of *LINC01612* in HEK293T cells, detected by immunofluorescent staining (D) and flow cytometric analysis (E) (n = 3 independent experiments). SBP-EGFP was used as an empty vector for GFP, and Nobody was used as positive micropeptide control. Scale bar, 100 μm.

(F) Western blot analysis showing the GFP protein levels of ORFs-EGFP in HEK293T cells.

(G) Expression of GFP and CXCR4 in ORF1/3 knockin HUES8-DE cells, detected by flow cytometric analysis. SBP-EGFP transfected HEK293T cells was used as a control.

(H) Immunofluorescent staining of pluripotency markers (OCT4 and SSEA4) in *LINC01612*-KD and control ESCs. Scale bar, 20 μm.

(I) Knockdown efficiencies of *LINC01612*-shRNAs in DE cells, examined by RT-qPCR (n = 3 independent experiments).

(J) The RNA levels of marker genes, including mesoderm genes (*MIXL1* and *T*) and pluripotency genes (*SOX2* and *OCT4*), in *LINC01612*-KD and control DE cells (n = 3 independent experiments).

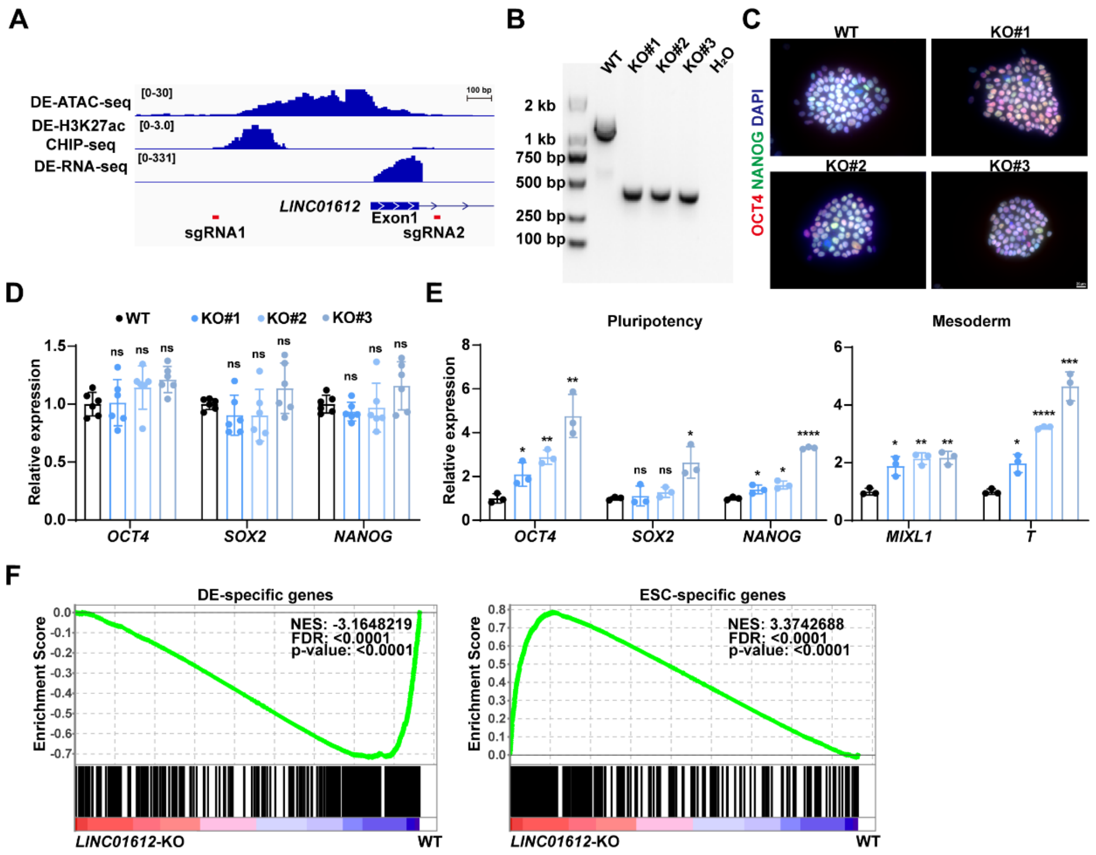

Figure S2. *LINC01612* is not essential for ESC maintenance but required for endoderm differentiation, related to Fig 2.

(A) Bedgraph for ATAC-seq, H3K27ac CHIP-seq and RNA-seq data in DE cells at *LINC01612* promoter loci.

(B) Genomic PCR showing the *LINC01612* deletion in *LINC01612*-KO ESCs.

(C) Immunofluorescent staining of pluripotency markers (OCT4 and NANOG) in wildtype and *LINC01612*- KO ESCs. Scale bar, 20 µm.

(D) The RNA levels of pluripotency genes (*OCT4*, *SOX2* and *NANOG*) in wildtype and *LINC01612*-KO ESCs (n = 6 independent experiments).

(E) The RNA levels of pluripotency genes (*OCT4*, *SOX2* and *NANOG*) and mesoderm genes (*MIXL1* and *T*) in wildtype and *LINC01612*-KO DE cells (n = 3 independent experiments).

(F) GSEA profile of DE-specific genes and ESC-specific genes in wildtype and *LINC01612*-KO DE cells.

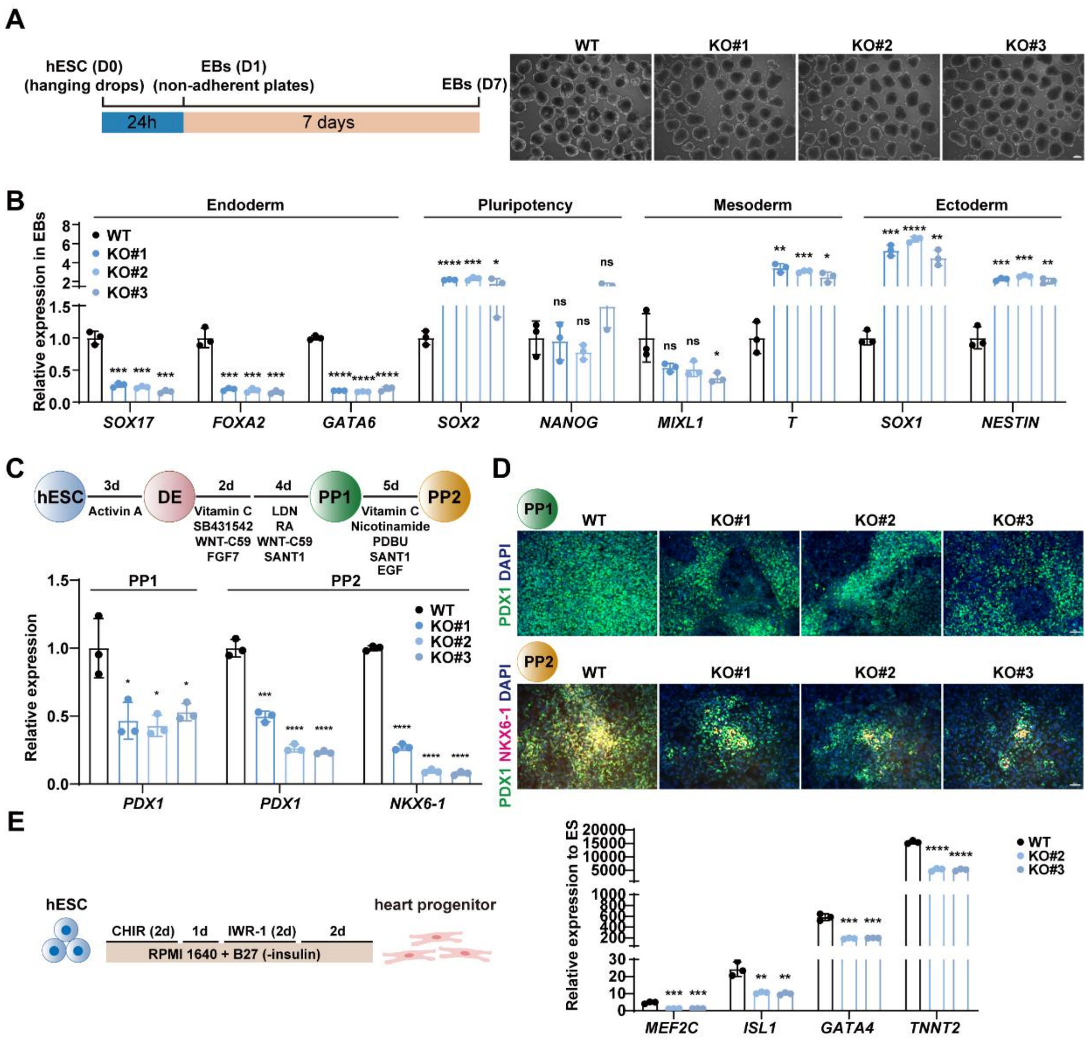

Figure S3. *LINC01612* is required for mesendodermal lineage differentiation, related to Fig 2.

(A) Left panel: schematic representation of EB differentiation of human ESCs over 7 days. Right panel: bright-field images of WT and *LINC01612*-KO EBs on day 7 in suspension culture, Scale bars, 200 µm.

(B) RT-qPCR analysis of pluripotency markers and lineage-specific markers (endoderm, mesoderm and ectoderm) in wildtype and *LINC01612*-KO EBs on day 7 (n = 3 independent experiments).

(C) Top panel: schematic representation of pancreatic differentiation of human ESCs. Bottom panel: the RNA levels of pancreatic progenitor markers in wildtype and *LINC01612*-KO cells at PP1 and PP2 (n = 3 independent experiments).

(D) Immunofluorescent staining of pancreatic progenitor markers in wildtype and *LINC01612*-KO cells at PP1 and PP2. Scale bars, 50 µm.

(E) Left panel: schematic representation of heart progenitor differentiation of human ESCs. Right panel: the RNA levels of heart progenitor markers in wildtype and *LINC01612*-KO cells (n = 3 independent experiments).

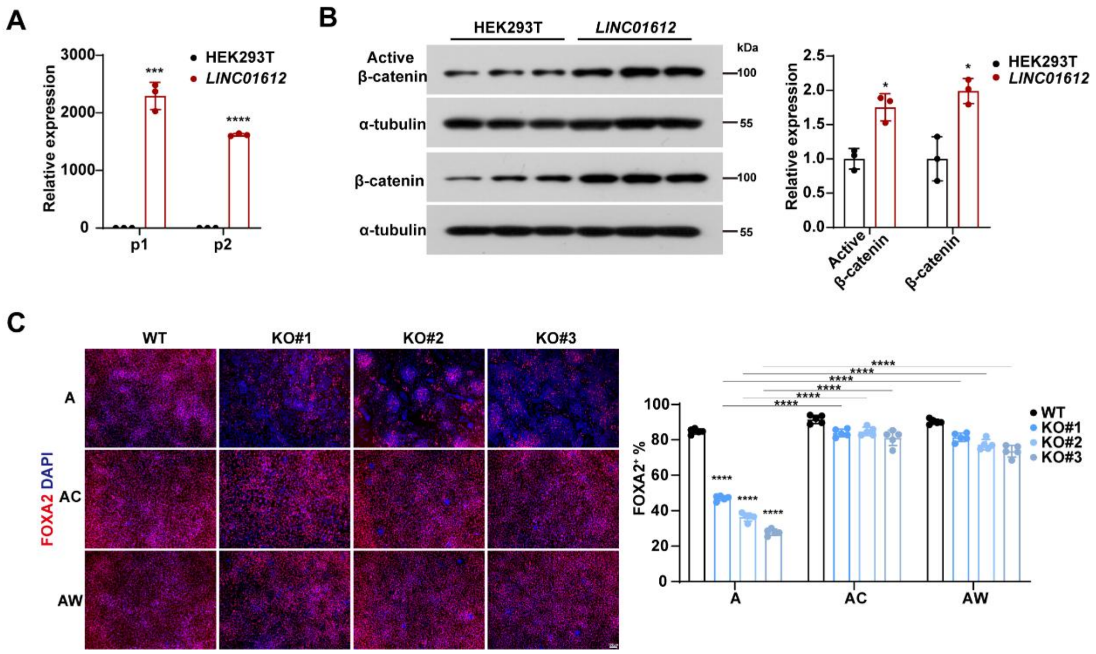

Figure S4. ***LINC01612*** **enhances WNT activity, related to Fig 3.**

(A) Efficient overexpression of *LINC01612* in HEK293T cells (n = 3 independent experiments), p1: primer 1; p2: primer 2.

(B) The levels of active β-catenin and total β-catenin in wildtype and *LINC01612*-overexpressing HEK293T cells. Quantitative results were shown on the right (n = 3 independent experiments).

(C) Immunofluorescent staining detection of FOXA2 in wildtype and *LINC01612*-KO DE cells treated with WNT activators during DE differentiation. Quantitative results were shown on the right (n = 6 images). Scale bar, 100 μm.

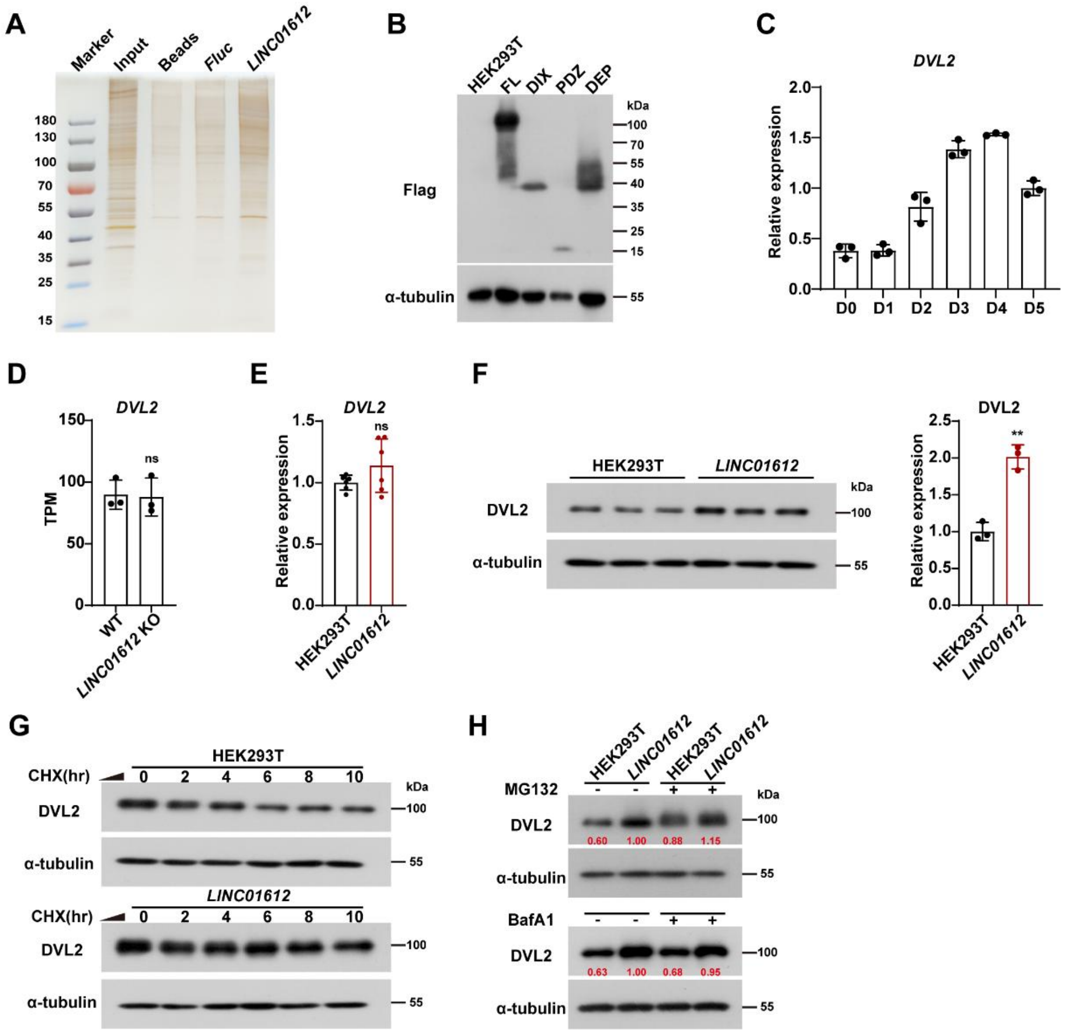

Figure S5. ***LINC01612*** **physically interacts with DVL2 protein and affects its protein stability, related to Fig 4.**

(A) The silver staining results of RNA pulldown in DE cells. Beads and *Fluc* (*Luciferase)* were used as negative controls.

(B) Western blot analysis showing the expression of Flag-tagged full-length DVL2 and its truncations expressed in HEK293T cells.

(C) Time course expression of *DVL2* during human endoderm differentiation detected by RT-qPCR (n = 3 independent experiments).

(D) The TPM value of *DVL2* in wildtype and *LINC01612*-KO DE cells (n = 3 independent experiments).

(E) The RNA level of *DVL2* in wildtype and *LINC01612*-overexpressing HEK293T cells (n = 6 independent experiments).

(F) The protein level of DVL2 in wildtype and *LINC01612*-overexpressing HEK293T cells. Quantitative results were shown on the right (n = 3 independent experiments).

(G) The endogenous protein level of DVL2 in wildtype and *LINC01612*-overexpressing HEK293T cells treated with CHX.

(H) The endogenous protein level of DVL2 in wildtype and *LINC01612*-overexpressing HEK293T cells treated with MG132 or BafA1.

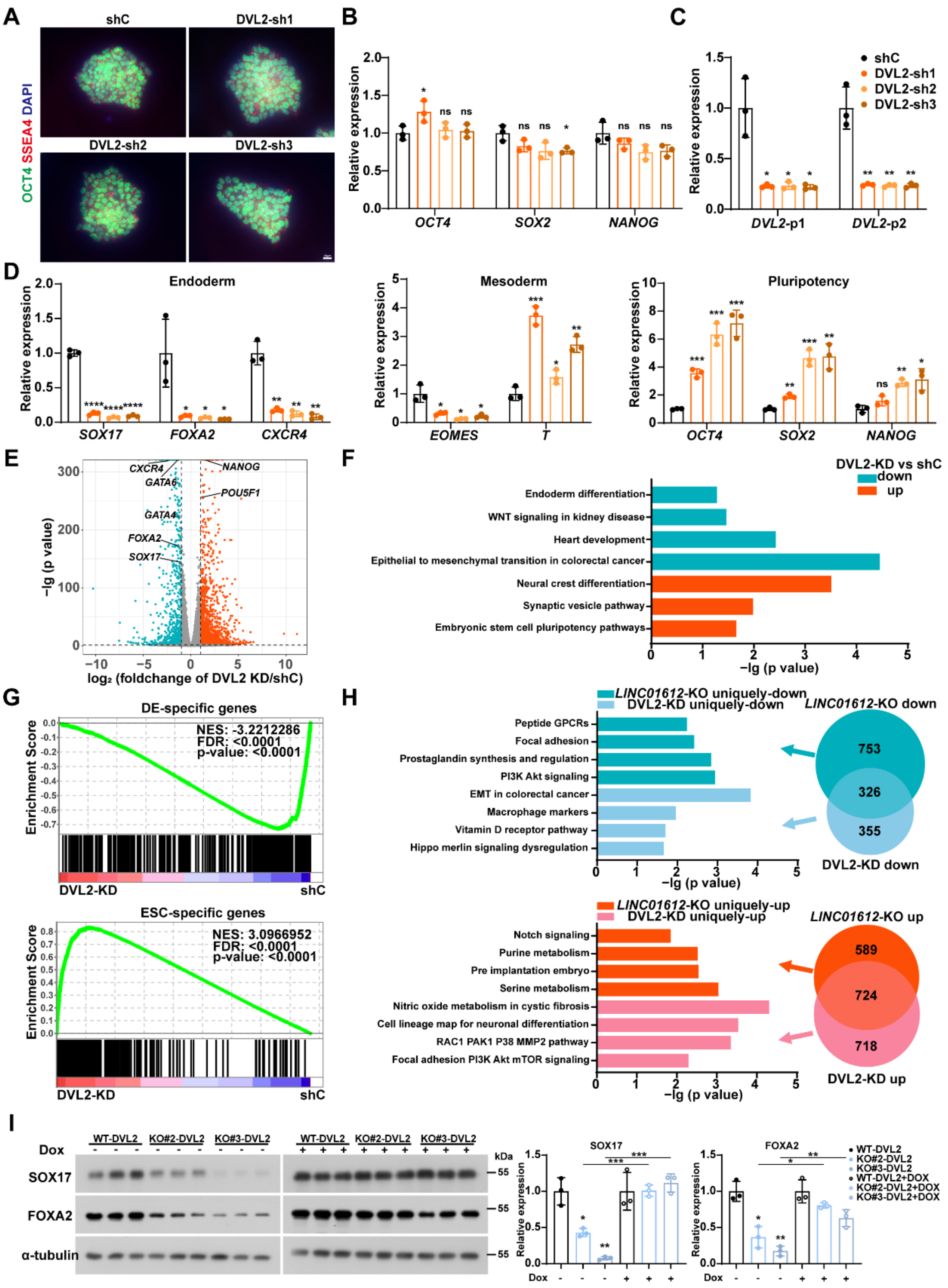

Figure S6. DVL2 functions in endoderm differentiation, related to Fig 5.

(A) Immunofluorescent staining of pluripotency markers (OCT4 and SSEA4) in shRNA control (shC) and DVL2-KD ESCs. Scale bar, 20 μm.

(B) The RNA levels of pluripotency genes (*OCT4*, *SOX2* and *NANOG*) in DVL2-KD and control ESCs (n = 3 independent experiments).

(C) Knockdown efficiencies of three *DVL2*-targeting shRNAs, determined by RT-qPCR in ESCs (n = 3 independent experiments).

(D) The RNA levels of endoderm genes (*SOX17*, *FOXA2* and *CXCR4*), mesoderm genes (*EOMES* and

T), and pluripotency genes (*OCT4*, *SOX2* and *OCT4*), in DVL2-KD and control DE cells (n = 3 independent experiments).

(E) Volcano plot showing differentially expressed genes identified by RNA-seq of control and DVL2-KD DE cells. Upregulated and downregulated genes upon DVL2-KD were shown in red and green, respectively.

(F) GO enrichment analysis of upregulated and downregulated genes in DVL2-KD DE cells, respectively.

(G) GSEA profile of DE-specific genes and ESC-specific genes in DVL2-KD and control DE cells.

(H) GO enrichment analysis of uniquely differentially expressed genes in *LINC01612*-KO and DVL2-KD DE cells.

(I) Western blot analysis showing the protein levels of SOX17 and FOXA2 in differentiated wildtype, *LINC01612*-KO and DVL2-overexpressing cells with *LINC01612*-KO. Quantitative results were shown at the bottom (n = 3 independent experiments).

Table S1. Sequences of two isoforms of *LINC01612*.

LINC01612 **isoform 1 sequences (5' to 3'): 1256bp**	GTGCTCTTGTGATTGTGACTGACACAGGCAGCACCCTTTCGCAGAAGT<br>AGATGTGCGTTGCTGAGAAATTTTCTGCCTAAGTTCTGGTTTTCTTTGC<br>AGCACCCAGCATTTGTTTCTAACACAGGGAAAGAGTGAGCTGTAAAAA<br>CCTCTCAAATTCAAGAAAGCGTAAAGCAAATTGAACAATACGGGTGAG<br>GAGCACGGACTATTCTCTCATGTTGAATGTGGCACTTTTTACATACAAG<br>CAGGAGAGCAACTACACCAAGATTTTGGAAAACATGTTATGTAAGGTT<br>AATTGTGACCTCTATGATGGGGATATGATAAAGAAGAAAAGTACCTCAC<br>AGCGATGATTGGCATGTGATCACTTAGCCAATGAGATAAGAGGCTGTAG<br>AAACGATGTGATAATGACATCTCCTGAGTTGCAGGTCCATATTAGAAGT<br>GGATATGAAGTAAACATTGAGCTTTCCTCACATTTAATTAACAAGCTTTA<br>TTATAGATACAGCTCAAATTAGTTAATTCAGGAAGCTTATTTTGTAGAAA<br>TTAAAACATTAATAAACACATACATTTAGAAATAAAGAACTCGGCTGAC<br>CCTTTGACTCTTTGAAACAAGTGGCCTTCCATGCTGTGAAAGATTGTAG<br>CCACTCTTTGAGACATTGGAATGGTGTCCATTGCATTTTATCAAGTTAAC<br>TATAGGTCAGCCTGGGAAATCTGAAGTTTCCTTATCTCCTGTCACTGTG<br>GTTGCTGTGAGATTTTGTGTCATCACTGTGCTGTCTTCCGTCTCCACATG<br>AAGGTGAGCTGTCTGGCTTCACTCTAACGTTTGTGATTTCACGCCCGAC<br>CTGATTGTTGCCAAGCAATTCCTTTGTTTGGAGAATTCTGTGCTTTGTTT<br>AGAATTTTTTAAAGGTTCTTTTCCTGTGGTCAGATTTCTGTTTGCTACTG<br>TGTCTTATGCCTTGTTCTCCTACAGAGAAGATTCTTTGAAGTTAGTGGA<br>AGCTCAGCTTAAAGGAAGATCTACTGCTCTGTGTGGTGGATGACCATG<br>GGGGAGCGAGAAGGCAGCCTCCGGCTTTTGTTTTGTGTGTGTGTGTGT<br>GTGTGTGTGTGATGTACCTGGAAAAATAACACGATTAGGATTAAAGAGT<br>ATGTTTGGCATGTTTAAAATAAATTGTAAACCAAACGTATTTTAAAGAAT<br>TTATACAAATTTGTCATTTAGTAAAATGAATGACTATTGAGTATTCATGTC<br>AATAAAACAACCACTTGAATACAACAAA
LINC01612 **isoform 2 sequences (5' to 3'): 1085bp**	GTGCTCTTGTGATTGTGACTGACACAGGCAGCACCCTTTCGCAGAAGT<br>AGATGTGCGTTGCTGAGAAATTTTCTGCCTAAGTTCTGGTTTTCTTTGC<br>AGCACCCAGCATTTGTTTCTAACACAGGGAAAGAGTGAGCTGTAAAAA<br>CCTCTCAAATTCAAGAAAGCGTAAAGCAAATTGAACAATACGGGTGAG<br>GAGCACGGACTATTCTCTCATGTTGAATGTGGCACTTTTTACATACAAG<br>CAGGAGAGCAACTACACCAAGATTTTGGAAAACATGTTATGTAAGGTT<br>AATTGTGACCTCTATGATGGGGATATGATAAAGAAGAAAAGTACCTCAC<br>AGCGATGATTGGCATGTGATCACTTAGCCAATGAGATAAGAGGCTGTAG<br>AAACGATGTGATAATGACATCTCCTGAGTTGCAGGTCCATATTAGAAGT<br>GGATATGAAGTAAACATTGAGCTTTCCTCACATTTAATTAACAAGCTTTA<br>TTATAGATACAGCTCAAATTAGTTAATTCAGGAAGCTTATTTTGTAGAAA<br>TTAAAACATTAATAAACACATACATTTAGAAATAAAGAACTCGGCTGAC<br>CCTTTGACTCTTTGAAACAAGTGGCCTTCCATGCTGTGAAAGATTGTAG<br>CCACTCTTTGAGACATTGGAATGGTGTCCATTGCATTTTATCAAGTTAAC<br>TATAGGTCAGCCTGGGAAATCTGAAGTTTCCTTATCTCCTGTCACTGTG<br>GTTGCTGTGAGATTTTGTGTCATCACTGTGCTGTCTTCCGTCTCCACATG<br>AAGAGAAGATTCTTTGAAGTTAGTGGAAGCTCAGCTTAAAGGAAGATC

	TACTGCTCTGTGTGGTGGATGACCATGGGGGAGCGAGAAGGCAGCCTC CGGCTTTTGTTTTGTGTGTGTGTGTGTGTGTGTGTGTGATGTACCTGGA AAAATAACACGATTAGGATTAAAGAGTATGTTTGGCATGTTTAAAATAA ATTGTAAACCAAACGTATTTAAAGAATTTATACAAATTTGTCATTTAGT AAAATGAATGACTATTGAGTATTCATGTCAATAAAACAACCACTTGAAT ACAACAAA

Table S2: The TPM of gene expression in wildtype, *LINC01612*-KO, shC and DVL2-KD DE cells. Provided in .xlsx file.

Table S3: The GO terms of the *LINC01612*-KO and DVL2-KD DEGs. Provided in .xlsx file.

Table S4: The mass spectrometry results of *LINC01612* or Fluc IP extracts. Provided in .xlsx file.

Table S5: Oligos used in this study.

Gene specific primers used in RACE assays and full-length cloning	
5-RACE-GSP1	GATTACGCCAAGCTTGTTTCAAAGAGTCAAAGGGTCAGCCGAG
3-RACE-GSP2	GATTACGCCAAGCTTGCAGAAGTAGATGTGCGTTGCTGAGAA
LINC01612-CDS-1256-F	GTGCTCTTGTGATTGTGACTGACA
LINC01612-CDS-1256-R	TTTGTTGTATTCAAGTGGTTGTTTTATTG
LINC01612-P1/2-F	GCACGGACTATTCTCTCATGTT
LINC01612-P1-R	AGGTTCTTTTCCTGTGGTCAGA
LINC01612-P2-R	CATGCCAAACATACTCTTTAATCCT
DVL2-CDS-2211-F	ATGGCGGGTAGCAGCACTGGGGGCGGT
DVL2-CDS-2211-R	CTACATAACATCCACAAAGAACTCGC
Fluc-F	AAGATGCCAAAAACATTAAGAAGG
Fluc-R	CACGGCGATCTTGCCGCCCTTCTT
LINC01612-1339-F	AACTCTGGGGACTCACAACACT
LINC01612-1339-R	CACTTACCTGGAATAAAGACTTGGG
Target gene	shRNA target sequence
LINC01612-sh1	GCAGGTCCATATTAGAAGT
LINC01612-sh2	GATTGGCATGTGATCACTT
Control (shC)	GAAGTATTCCGCGTACGTT
DVL2-sh1	GGGTGGTGAAGGAAGAAATTT
DVL2-sh2	AGCGTCACAGATTCCACAATG
DVL2-sh3	GCCCACTTTCTCCTACCAATA
Target gene	sgRNAs target sequence
LINC01612-KO-g1	ATTATGTATCCAAATCATCG
LINC01612-KO-g2	AAGCTAAGATGAGTAGGCTA
LINC01612-ORF1-K1-g1	ACATGCCAATCATCGCTGTG
LINC01612-ORF3-KI-g1	AAGTACCTCACAGCGATGAT
Gene name	qPCR primers
NANOG	F: GAGACTTGGCACGCCTGT
	R: CCAGGTTGAATTGTTCCAGGTC
OCT4	F: CCCCAGCCTTTACTCTTCCTA
	R: CCAGGTTGAATTGTTCCAGGTC

SOX2	F: CAAAGCAGAAACCCTCGTGC
	R: TCTCACTCGGTTCTCGATACTG
MIXL1	F: GTCATTTGCTGTGGGTGATG
	R: AGAAAAACGAGGGAAATGGG
EOMES	F: CACATTGTAGTGGGCAGTGG
	R: CGCCACCAAACTGAGATGAT
T	F: GAGACTTGGCACGCCTGT
	R: GGTACCCCGACATCCACTT
SOX17	F: GATGATCGTGACCAAGAACGG
	R: CCACGAAGTCCAGCAGGAA
FOXA2	F: GCATGACTCCGGTGTGAATCT
	R: TCACACGTCAGGATAGTTGCAGT
GATA4	F: GGAGCAGCTACTATGCAGAGC
	R: CGTGTTCATGCCGTTCATCC
GATA6	F: CAGGCGTTGCACAGATAGTG
	R: CCCGACACCCCAATCTC
CXCR4	F: AGTTCCTACGCTTCGCATCCCTTC
	R: TGAACAGCAGCAAGTCCTCCCA
GAPDH	F: TACACCGAGGAAATGGGCTCA
	R: AGATGATGGAGTAGATGGTGGG
MALAT1	F: AATGAAGGGGTCATTGATGG
	R: AAGGTGAAGGTCGGAGTCAA
LINC01612-p1	F: GCAGGAGAGCAACTACACCAA
	R: GCTAAGTGATCACATGCCAATC
LINC01612-p2	F: CTGTGCTGTCTTCCGTCTCC
	R: TCGGGCGTGAAATCACAAAC
LINC01612-p3	F: AGGGAAAGAGTGAGCTGTAAAAAC
	R: TCACCCGTATTGTTCAATTTGCTTT
DVL2-p1	F: TCAGCAGCGTCACAGATTCC
	R: GTCTCCCCGCTCATTGCTC
DVL2-p2	F: GAGGAAGAGACTCCCTACCTG
	R: CGGGCGTTGTCATCTGAAAT
PDX1	F: CCTTTCCCATGGATGAAGTC
	R: GAACTCCTTCTCCAGCTCTA
NKX6-1	F: AGACCCACTTTTTCCGGACA
	R: CCAACGAATAGGCCAAACGA
MEF2C	F: CTGGTGTAACACATCGACCTC
	R: GATTGCCATACCCGTTCCCT
ISL1	F: GCAGAGTGACATAGATCAGCCTG
	R: GCCTCAATAGGACTGGCTACCA
TNNT2	F: AAGAGGCAGACTGAGCGGGAAA
	R: AGATGCTCTGCCACAGCTCCTT

Supplemental Methods

Quantitative RT-qPCR and droplet digital PCR

Total RNA was extracted from cultured cells using the Hipure Total RNA Mini Kit (Magen, Cat#R4111-03) or TriPure Isolation Reagent (Roche, Cat#11667165001) following the manufacturer's protocols. cDNA was synthesized from 1 μg of total RNA using the ABScript II RT Master Mix (ABclonal, Cat#RK20402). Gene expression was quantified using 2×SYBR Green Fast qPCR Mix (ABclonal, Cat#RK21203) on a CFX384 qPCR system (Bio-Rad), with *GAPDH* as the internal control for normalization. The primers used in all RT-qPCR assays were listed in Table S5.

The copy numbers of *LINC01612* were quantified by ddPCR outsourced to Guangzhou Forevergen Biosciences. Briefly, the 20 μL PCR reaction mixture consisted of 1.8 μL forward primer, 1.8 μL reverse primer, 4.4 μL H_2O, 2 μL cDNA, and 10 μL ddPCR Supermix. Droplet generation was performed using MicroDrop-100A. 40 μL of the emulsion was then transferred into the 96-well PCR plate, which was then sealed with pierceable heat-seal film using a preheated heat sealer. The plate was cycled to an endpoint as per the manufacturer's protocol, then placed in the MicroDrop-100B biochip reader for detection and analyzed with QuantDrop software.

Flow cytometry

DE cells were dissociated with TrypLE (Gibco, Cat#12604021) and washed with DPBS containing 2% FBS. Cells were incubated with CD184-APC (BD, Cat#555976) for 30 minutes, fixed with Transcription Factor Buffer Set (BD, Cat#562574), and stained with SOX17-Alexa 488 (BD, Cat#562205). Isotype control was used. $SOX17^+$ or $CXCR4^+$ cells were analyzed using a flow cytometer (ACEA NovoCyte or CytoFLEX), with data analyzed by FlowJo software.

Immunofluorescence staining

Cells were fixed with 4% paraformaldehyde following PBS washes, then permeabilized and blocked using blocking buffer containing 10% donkey serum and 0.3% Triton X-100. Afterward, cells were incubated overnight at 4°C with primary antibodies at the appropriate dilution. The antibodies used included: SOX17 (R&D, Cat#AF1924, 1:200), FOXA2 (HuaBio, Cat#ET1703-76, 1:200), OCT4 (CST, Cat#2750, 1:200), SOX2 (BD, Cat#561469, 1:200), and SSEA4 (CST, Cat#4755, 1:200). After three PBS washes, the cells were incubated with secondary fluorescent antibodies. The cells were then incubated with DAPI solution for 5-10 minutes at room temperature, protected from light. Images were captured and analyzed using an Olympus IX53 microscope.

Western blot

Cell pellets were lysed in RIPA buffer (Beyotime, Cat#P0013C) with protease inhibitors (Roche, Cat#4693132001) for 30 minutes at 4°C. After centrifugation, supernatants were collected, and proteins were separated by 10% SDS-PAGE and transferred to nitrocellulose membranes (Millipore, Cat#Z746010). The membranes were washed with TBST, blocked with 5% skimmed milk in TBST for 30 minutes, and incubated with primary antibodies overnight at 4°C or for 2 hours at room temperature. After washing, the membranes were incubated with HRP-conjugated secondary antibodies for 1 hour. Signals were detected using ECL (Millipore, Cat#WBUSLS0100) and a film imaging system. Primary antibodies included: SOX17 (R&D, Cat#AF1924, 1:1000), FOXA2 (R&D, Cat#AF2400, 1:1000), GAPDH (Proteintech, Cat#10494-1-AP, 1:5000), α-Tubulin (Proteintech, Cat#11224-1-AP, 1:5000), active β-catenin (CST, Cat#8814, 1:1000), β-catenin (CST, Cat#8480, 1:1000), DVL2 (Proteintech, Cat#12037-1-AP, 1:1000), GFP (SANTA CRUZ, Cat#sc-101536, 1:1000) and Flag (Sigma, Cat# F1804, 1:1000).

RACE and cDNA cloning

After 3 days of DE differentiation, HUES8 cells were harvested, and total RNA was isolated using the Hipure Total RNA Mini Kit (Magen, Cat#R4111-03). The 5' and 3' regions of *LINC01612* were amplified with the SMARTer RACE 5'/3' kit (TAKARA, Cat#634858) according to the manufacturer's instructions. The resulting PCR products were then cloned and sequenced by Sanger sequencing. Gene-specific primers, along with those used to clone the full-length *LINC01612* transcripts, were provided in Table S5.

Cytosolic/nuclear fractionation

Approximately 1 million DE cells were collected by TrypLE and re-suspended in DPBS. Cells were lysed in CE buffer (10 mM Hepes, 60 mM KCl, 1 mM EDTA, 0.34 M sucrose, 0.3% NP-40, 1 mM DTT) with inhibitors on ice for 10 minutes, then centrifuged at 3000 rpm for 10 minutes to separate nuclear and cytoplasmic fractions. RNA was extracted using TriPure reagent (Roche, Cat#11667165001) and analyzed by RT-qPCR.

RNA pull-down

In brief, biotin-labeled *luciferase (Fluc)*, *LINC01612* and truncated *LINC01612* fragments were transcribed *in vitro* using HiScribe T7 High Yield RNA Synthesis Kit (NEB, Cat#E2040S) and biotin-16-UTP (Roche, Cat#11388908910) according to the manufacturer's instructions. A total of 10 μg of biotin-labeled *LINC01612* or truncated *LINC01612* were incubated with whole-cell lysates from DE cells or HEK293T cells. Subsequently, 40 μL of Dynabeads MyOne Streptavidin C1 (Invitrogen, Cat#65001) was used to capture RNA-protein complexes. These complexes were then analyzed by silver staining, mass spectrometry (provided by Institute of Hydrobiology, Chinese Academy of Science), and Western blot.

RNA immunoprecipitation (RIP)

RIP assays were carried out using the Magna RNA-binding protein immunoprecipitation kit (Millipore, Cat#17–700) according to the manufacturer's protocol. Cell lysates were incubated overnight at 4 °C with Protein A/G Magnetic Beads (MCE, Cat#HY-K0202) and the appropriate antibodies. RNA purification was achieved using proteinase K treatment, followed by extraction with TRIzol (Invitrogen, Cat#10296010) and phenol/chloroform/isoamyl alcohol. Quantitative analysis of the purified RNA was then performed using RT-qPCR.

Co-immunoprecipitation

The Co-IP experiment was mainly conducted based on the published experimental method (Ponzielli et al., 2013). Cells cultured on 10-cm dish were washed with PBS and lysed using IP WCE buffer (25 mM Tris pH 7.6, 0.5% NP-40, 250 mM NaCl, 3 mM EDTA, 3 mM EGTA, 1× protease inhibitor cocktail, 1 mM PMSF, and 0.5 mM DTT) on ice. After centrifugation to clarify the cell lysate, the supernatant was incubated with 2.5 μg of DVL2 or IgG antibody at 4 °C overnight with rotation, followed by an additional 3 hours' incubation with Protein A/G Magnetic Beads at 4 °C. The immunoprecipitates were washed with IP Wash Buffer (25 mM Tris pH 7.6, 1% NP-40, 200 mM NaCl, 3 mM EDTA, and 3 mM EGTA), then resuspended in 40 μL of 1× SDS loading buffer. The samples were analyzed by Western blot.

CHX, MG132 and BafA1 treatments

DE cells and HEK293T cells were treated with CHX (MCE, Cat#HY-12320) and collected at different time points. Similarly, cells were treated with MG132 (MCE, Cat#HY-13259) or BafA1 (MCE, Cat#HY-100558) followed by collection at 0- and 6-hour post-treatment. Proteins were subsequently extracted and analyzed by Western blot.

REFERENCES

Ponzielli, R., Tu, W.B., Jurisica, I., and Penn, L.Z. (2013). Identifying Myc interactors. Methods Mol Biol *1012*, 51-64. 10.1007/978-1-62703-429-6_4.
